# *Xanthomonas campestris* VemR enhances the transcription of the T3SS key regulator HrpX via physical interaction with HrpG

**DOI:** 10.1111/mpp.13293

**Published:** 2023-01-10

**Authors:** Rui‐Fang Li, Jian‐Ling Peng, Qian‐Qian Liu, Zheng Chang, Yi‐Xin Huang, Ji‐Liang Tang, Guang‐Tao Lu

**Affiliations:** ^1^ State Key Laboratory for Conservation and Utilization of Subtropical Agro‐Bioresources College of Life Science and Technology, Guangxi University Nanning China; ^2^ Guangxi Key Laboratory of Biology for Crop Diseases and Insect Pests Plant Protection Research Institute, Guangxi Academy of Agricultural Sciences Nanning China

**Keywords:** HrpG activity, single‐domain response regulator, two‐component system, type III secretion system, *Xanthomonas campestris*

## Abstract

VemR is a response regulator of the two‐component signalling systems (TCSs). It consists solely of a receiver domain. Previous studies have shown that VemR plays an important role in influencing the production of exopolysaccharides and exoenzymes, cell motility, and virulence of *Xanthomonas campestris* pv. *campestris* (Xcc). However, whether VemR is involved in the essential pathogenicity determinant type III secretion system (T3SS) is unclear. In this work, we found by transcriptome analysis that VemR modulates about 10% of Xcc genes, which are involved in various cellular processes including the T3SS. Further experiments revealed that VemR physically interacts with numerous proteins, including the TCS sensor kinases HpaS and RavA, and the TCS response regulator HrpG, which directly activates the transcription of HrpX, a key regulator controlling T3SS expression. It has been demonstrated previously that HpaS composes a TCS with HrpG or VemR to control the expression of T3SS or swimming motility, while RavA and VemR form a TCS to control the expression of flagellar genes. Mutation analysis and in vitro transcription assay revealed that phosphorylation might be essential for the function of VemR and phosphorylated VemR could significantly enhance the activation of *hrpX* transcription by HrpG. We infer that the binding of VemR to HrpG can modulate the activity of HrpG to the *hrpX* promoter, thereby enhancing *hrpX* transcription. Although further studies are required to validate this inference and explore the detailed functional mechanism of VemR, our findings provide some insights into the complex regulatory cascade of the HpaS/RavA‐VemR/HrpG‐HrpX signal transduction system in the control of T3SS.

## Graphical abstract

VemR, a single‐domain response regulator, modulates the expression of type III secretion system via binding to the key regulator HrpG in *Xanthomonas campestris* pv. *campestris*.

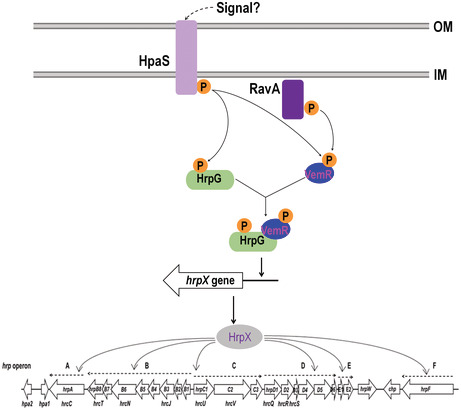

## 1 INTRODUCTION

Bacteria have developed various regulatory mechanisms to precisely control the expression of varied genes involved in different physiological processes in response to environmental changes. Two‐component signalling systems (TCSs) are a major mechanism widely adopted by bacteria to sense environmental changes and modulate subsequently the expression of related genes (Buschiazzo & Trajtenberg, 2019; Gao et al., 2019). TCSs are composed of at least a histidine kinase (HK) sensor and a response regulator (RR). Generally, a membrane‐associated HK sensor carries out autophosphorylation on a conserved histidine residue in the transmitter domain on sensing a specific environmental stimulus. The phosphoryl group is then transferred to the conserved aspartate residue in the receiver (REC) domain of a cognate RR, leading to a conformational change and the activation of its output domain, which in turn regulates the downstream targets (Buschiazzo & Trajtenberg, 2019; Gao et al., 2019). Notably, some RRs contain only a phosphoryl‐accepting REC domain but lack a dedicated output domain. Genomic analysis reveals that these RR proteins, referred to as single‐domain response regulators (SD‐RRs), are widespread in bacteria, constituting the second largest class of RR proteins (about 23%) (Gao et al., 2019). Given that the SD‐RRs do not have an output domain such as a DNA‐binding domain, they are thought to act by directly interacting with their downstream protein targets and allosterically regulating the activity of the targets. However, only a few SD‐RRs have been studied for their cellular functions. So far, the best characterized SD‐RR is the chemotactic protein CheY. The *Escherichia coli* CheY and its homologues in a number of other bacteria have been shown to regulate cell swimming motility via protein–protein interaction. When CheY is phosphorylated by the phosphoryl group from the phosphorylated HK sensor CheA, the phosphorylated CheY binds to FliM and FliN, the components of the flagellar motor switch complex, to modulate the flagellar motor rotation (Parkinson et al., 2015; Sarkar et al., 2010).

*Xanthomonas campestris* pv. *campestris* (Xcc) is a gram‐negative bacterial phytopathogen that infects almost all of the cruciferous plants and causes a threatening disease (black rot) to brassica crops worldwide (Vicente & Holub, 2013). The virulence of Xcc toward hosts depends on a number of pathogenicity factors, including lipopolysaccharides, exopolysaccharides, exoenzymes (such as amylase, cellulase, protease, and pectate lyase) secreted by the type II secretion system (T2SS), effectors (T3Es) secreted by the type III secretion system (T3SS), cell motility, and biofilm (An et al., 2020). The *Xanthomonas* T3SSs and their regulation have been subjected to extensive studies. It is clear that, like many other *Xanthomonas* T3SSs, the Xcc T3SS apparatus is encoded by the *hrp* (hypersensitive response and pathogenicity) gene cluster, which consists primarily of six operons (*hrpA–hrpF*) and more than 20 *hrp* or *hrc* (*hrp*‐conserved) genes (Arlat et al., 1991; Gürlebeck et al., 2006; Huang et al., 2009; Qian et al., 2005). The activation of Xcc *hrp/hrc* genes is mainly controlled by three key regulators, HpaS, HrpG, and HrpX (Alvarez‐Martinez et al., 2020; An et al., 2020). Disruption of *hpaS*, *hrpG* or *hrpX* in Xcc resulted in loss of virulence in host plants and hypersensitive response (HR) in nonhost plants (Huang et al., 2009; Li et al., 2014). HrpX is an AraC‐type transcriptional regulator. It induces the transcription of the *hrp*/*hrc* genes and some T3E‐encoding genes by directly binding to their promoters (Huang et al., 2009; Koebnik et al., 2006; Wengelnik & Bonas, 1996). HpaS is a membrane‐bound HK sensor. It forms a TCS with HrpG, an OmpR family DNA‐binding regulator, which activates the expression of *hrpX* (Li et al., 2014).

Interestingly, in addition to HrpG, HpaS can also co‐opt the SD‐RR VemR to form another TCS (Li et al., 2020). VemR is involved in extracellular polysaccharide (EPS) production, cell motility, and virulence of Xcc (Tao & He, 2010). It has been shown that after being phosphorylated by HpaS or RavA, VemR directly interacts with the flagellum rotor protein FliM or the flagellum biosynthesis regulator FleQ to control cell motility (Li et al., 2020; Lin et al., 2022). Recently, we found that the Xcc VemR is a global regulator that plays an important role in modulating the expression of the T3SS. Here, we demonstrate that VemR positively regulates the expression of the *hrp* gene cluster via HrpG and present evidence showing that VemR physically interacts with HrpG and enhances the transcriptional activation of *hrpX* by HrpG.

## 2 RESULTS

### 2.1 VemR is a global regulator modulating the expression of a large number of genes involved in various cellular processes

**FIGURE 1:**
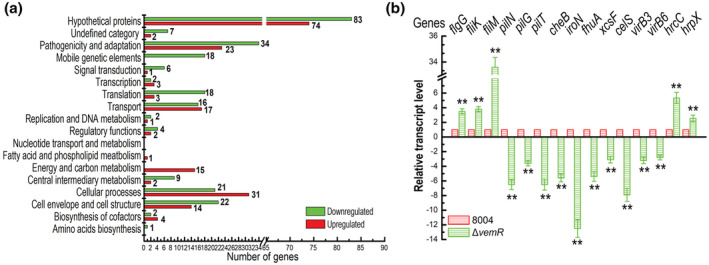
VemR acts as a global regulator affecting a number of genes involved in a variety of cellular processes cultured in nutrient‐yeast‐glycerol (NYG) medium. (a) Functional categories of the 438 differentially expressed genes (DEGs) in the *vemR*‐deletion mutant of *Xanthomonas campestris* pv. *campestris* (Xcc). The transcriptomes of Xcc strains cultured in NYG medium were investigated by RNA‐Seq. Among the 438 DEGs, 193 and 245 were up‐regulated and down‐regulated, respectively. These genes were broadly categorized according to their biological function. (b) Reverse transcription‐quantitative PCR assay of the expression level of several virulence‐related genes regulated by VemR in Xcc strains. RNA was isolated from the cultures of the Xcc wild‐type strain 8004 and the *vemR* deletion mutant Δ*vemR* grown in NYG medium to a concentration of OD_600_ of 0.6. Relative gene expression with respect to the corresponding transcript levels in the wild‐type strain was calculated. Values given are the means ± *SD* of triplicate measurements from a representative experiment. Differences were evaluated using Student's *t* test (**p < 0.01). Genes were considered to be differentially expressed if |log_2_ (fold change)| ≥ 1 compared to the wild type. Similar results were obtained in two other independent experiments

VemR is encoded by the open reading frame (ORF) *XC_2252* in the genome of the Xcc wild‐type strain 8004 (Qian et al., 2005). Previous studies showed that deletion of *vemR* resulted in a severe reduction in virulence, EPS production, and cell motility (Li et al., 2020; Tao & He, 2010). To gain a better understanding of the regulatory role of VemR in various cellular processes of Xcc, we determined the transcriptome of the *vemR* deletion mutant strain Δ*vemR* by RNA‐sequencing (RNA‐Seq). The mutant strain as well as the wild‐type strain 8004 (Table S1) were grown to the mid‐exponential phase (OD_600_ = 0.6) in nutrient‐yeast‐glycerol (NYG), a medium widely used in physiologic studies of Xcc (Daniels et al., 1984). Total RNA was extracted from two independent biological replicates for each strain and analysed by RNA‐Seq as described previously (Cui et al., 2018). The results displayed that out of the 4617 annotated protein‐coding genes in the genome of the Xcc strain 8004 (Luneau et al., 2022; Qian et al., 2005), 438 were differentially expressed by two‐fold or more in the *vemR* deletion mutant (Table S2). Among these 438 differentially expressed genes (DEGs), 193 were up‐regulated and 245 were down‐regulated (Figure 1a and Table S2). To verify the transcriptome changes, 15 DEGs related to virulence and adaption were selected to quantify their expression level using reverse transcription‐quantitative real‐time PCR (RT‐qPCR). The total RNAs used for RT‐qPCR were prepared from the bacterial cells grown in the same conditions as those for RNA‐Seq. The RT‐qPCR results showed that the transcriptional levels of all the tested genes were obviously changed in the mutant Δ*vemR* compared to the wild type, and the expression patterns of these selected genes are all consistent with that observed in the data obtained from the transcriptome analysis (Figure 1b and Table S2).

To get an insight into the functions of the genes regulated by VemR, we conducted a functional clustering analysis according to the genome annotation of the Xcc strain 8004 (He et al., 2007; Qian et al., 2005). Based on the clusters of orthologous groups (COG), out of the 438 DEGs in Δ*vemR* mutant, 272 were assigned to 15 various functional categories and 166 were predicted to encode hypothetical proteins or proteins that have not been given a functional category (Figure 1a and Table S2). The dominant functional categories are “pathogenicity and adaption” and “cellular processes”. In total, 57 and 52 genes were assigned to these two categories, respectively (Figure 1a). Notably, 105 genes were assigned to “cell envelope and cell structure” (36), “transport” (33), “translation” (21), and “energy and carbon metabolism” (15) (Figure 1a).

Consistent with the finding that VemR regulates cell motility (Li et al., 2020; Lin et al., 2022; Tao & He, 2010), the identified transcriptional profiles reveal that VemR has a crucial impact on a number of genes that contribute to cell motility. All of the 52 DEGs assigned to the category “cellular processes” are related to chemotaxis and flagella‐dependent motility (Table S2). Additionally, out of the 22 down‐regulated genes assigned to cell envelope and cell structure, 18 (*XC_0937*, *XC_0938*, *XC_0939*, *XC_0940*, *XC_0941*, *XC_1059*, *XC_1183*, *XC_1184*, *XC_1185*, *XC_1186*, *XC_1187*, *XC_1358*, *XC_1359*, *XC_1621*, *XC_1622*, *XC_1624*, *XC_1626*, and *XC_3823*) are involved in the type IV pilus biogenesis/fimbrial assembly. Given that in xanthomonads and other plant‐pathogenic bacteria the type IV pili were reported to play a role in cell motility (Köhler et al., 2000; Mhedbi‐Hajri et al., 2011), the motility deficiency in the Δ*vemR* mutant might be partially due to the reduced pilus biogenesis. It has been shown that the mutant Δ*vemR* produced less type II‐secreted plant cell wall‐degrading enzymes, such as extracellular amylase, cellulase, and protease, relative to the wild‐type strain (Tao & He, 2010). Scanning revealed that the DEGs assigned to the category “pathogenicity and adaption” include two genes (*XC_0741* and *XC_0742*) encoding T2SS components and eight genes (*XC_0125*, *XC_0126*, *XC_0783*, *XC_1120, XC_2483, XC_3379, XC_3590*, and *XC_3591*) encoding extracellular amylase, cellulase or protease, which were all down‐regulated in *vemR* mutant (Table S2). This result supports that VemR also plays a role in the regulation of exoenzyme production in Xcc.

Importantly, in addition to the genes involved in bacterial cell motility and T2SS‐secreted enzyme production, which are known to be regulated by VemR, the transcriptome analysis uncovered that VemR also modulates the expression of genes encoding the T3SS, T4SS (type IV secretion system), and TonB‐dependent receptors. The T3SS‐related DEGs include 11 *hrp/hrc* genes, namely, *XC_3002/hpa1*, *XC_3003/hrcC*, *XC_3004/hrcT*, *XC_3005/hrpB7*, *XC_3006/hrcN*, *XC_3008/hrpB4*, *XC_3009/hrcJ*, *XC_3011/hrpB1*, *XC_3019/hrpD5*, *XC_3020/hrpD6*, and *XC_3076*/*hrpX* (Table S2). As mentioned above, the T3SS is critical for Xcc pathogenicity. Interestingly, under the test conditions, these DEGs were up‐regulated in the mutant strain Δ*vemR* compared to the wild‐type strain 8004 (Table S2). The T4SS‐related DEGs include 10 *vir* genes (*XC_1027*/*virB6*, *XC_1632*/*virB8*, *XC_1633*/*virB9*, *XC_1634*/*virB10*, *XC_1635*/*virB11*, *XC_1636*/*virB1*, *XC_1637*/*virB2*, *XC_1638*/*virB3*, *XC_1639*/*virB4*, and *XC_2016*/*virB6*) (Table S2). All of these *vir* genes were down‐regulated in the *vemR* mutant. The T4SS is a large multiprotein nanomachine composed of a core set of 12 different proteins (VirB1 to VirB11 plus VirD4) in many gram‐negative bacterial species. Xanthomonads use the T4SS to kill other bacterial cells in a contact‐dependent manner, thereby obtaining a growth advantage in interbacterial competition (Souza et al., 2015). Fourteen of the DEGs encode TonB‐dependent receptors: *XC_0124*, *XC_0756*, *XC_0759*, *XC_1165*, *XC_1222*, *XC_1241*, *XC_1546*, *XC_1644*, *XC_2296*, *XC_2484*, *XC_2485*, *XC_2983*, *XC_3209*, and *XC_0988* (Table S2). It has been demonstrated that in *Xanthomonas* spp. TonB‐dependent receptors are involved in efficient nutrition uptake for bacterial growth under in planta infection conditions (Blanvillain et al., 2007).

However, the DEGs do not harbour any *gum* genes that code for EPS production. This observation is inconsistent with the previous finding that deletion of *vemR* significantly reduced EPS production (Li et al., 2020; Tao & He, 2010). In Xcc, the *gum* cluster consisting of 12 genes (*gumB* to *gumM*) is responsible for the assembly, polymerization, and export of EPS (Ielpi et al., 1993; Katzen et al., 1998). It has been demonstrated that in Xcc EPS synthesis is initiated in the late‐exponential growth phase and reaches maximal production during the stationary growth phase, and that the expression of the *gum* genes mirrors the time course of EPS production (Harding et al., 1995; Vojnov et al., 2001). As described above, our transcriptome analysis used the bacterial cells in the mid‐exponential growth phase. Therefore, this inconsistency is probably due to the employment of bacterial cells from different growth phases in the experiments.

Taken together, the results from the transcriptome analysis reveal that VemR has a broader regulatory role than previously observed. It acts as a global regulator involved in many cellular processes in Xcc.

### 2.2 VemR positively regulates *hrp* gene expression and HR induction in planta

**FIGURE 2:**
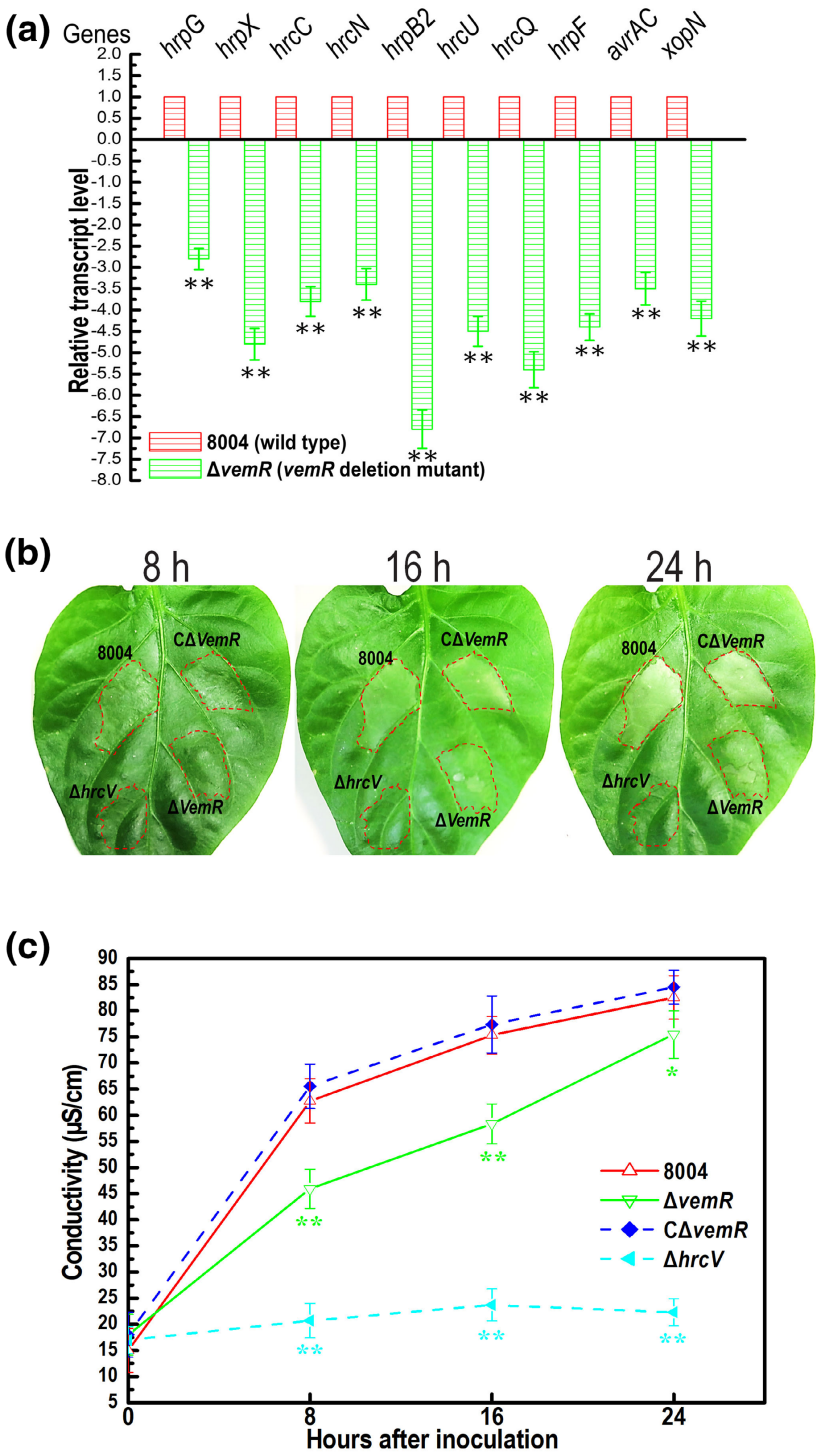
VemR plays a role in *Xanthomonas campestris* pv. *campestris* (Xcc) type III secretion system (T3SS). (a) The transcription level of T3SS‐related genes in *vemR* deletion mutant was estimated by reverse transcription‐quantitative PCR. Xcc strains were grown in XVM2 medium to a concentration of OD_600_ of 0.6, and RNA was isolated. Relative gene expression with respect to the corresponding transcript levels in the wild‐type strain 8004 was calculated. Values given are the means ± *SD* of triplicate measurements from a representative experiment. Differences were evaluated using Student's *t* test (**p < 0.01). Similar results were obtained in two other independent experiments. (b) Hypersensitive response (HR) symptoms observed on pepper leaves after infiltration. Bacterial cells from overnight cultures of the Xcc wild‐type strain (8004), Δ*vemR*, CΔ*vemR*, and Δ*hrcV* (negative control) were collected, washed with, and resuspended in 10 mM sodium phosphate buffer to a density of 10^7^ cfu/ml. Approximately 5 μl of bacterial resuspension was infiltrated into the leaf mesophyll tissue with a blunt‐end plastic syringe, then the plants were placed under a bright light for 24 h. The HR symptoms caused by Xcc strains were recorded at 8, 16, and 24 h postinoculation (hpi). Three replications were done in each experiment, and each experiment was repeated three times. The results presented are from a representative experiment, and similar results were obtained in all other independent experiments. (c) Electrolyte leakage from pepper leaves inoculated with Xcc strains. The conductivity of the infiltration spots was measured at 0, 8, 16, and 24 hpi, with four 0.4 cm^2^ leaf disks collected from the infiltrated area for each sample. Three samples were taken for each measurement in each experiment. Data are shown as the mean ± *SD* of three replicates from a representative experiment, and similar results were obtained in two other independent experiments. Statistical differences denoted by asterisks at data points were determined by analysis of variance and Dunnett's post hoc test for comparison to the wild type at each time point. *p < 0.05, **p < 0.01

Our transcriptomic data show that the *hrp* genes were up‐regulated in the mutant Δ*vemR* cultured in the nutrition‐rich medium NYG, suggesting that VemR negatively affects the expression of *hrp* genes under the test conditions. To clarify whether VemR regulates *hrp* gene expression in minimal medium in the same manner as in nutrition‐rich medium, we determined by RT‐qPCR analysis the transcript levels of several operons in the *hrp* cluster (*hrcC*, *hrcN*, *hrpB2*, *hrcU*, *hrcQ*, and *hrpF*), two key regulators (*hrpX* and *hrpG*), and two T3E genes (*avrAC* and *xopN*) in the mutant Δ*vemR* cultured in XVM2, a minimal medium that mimics the nutrition environment in plant tissues (Astua‐Monge et al., 2005). The results displayed that the expression of all of the tested genes was significantly down‐regulated in the Δ*vemR* mutant compared to the wild type (Figure 2a), indicating that VemR positively controls the expression of T3SS in minimal medium.

The influence of VemR on the induction of HR in a nonhost plant was also evaluated. Bacterial suspensions of the Δ*vemR* mutant and other three strains (the wild type 8004, the complemented strain CΔ*vemR*, and the *hrcV* deletion mutant Δ*hrcV*) with a concentration of 10^7^ cfu/ml were introduced into the leaves of the nonhost plant pepper (*Capsicum annuum*) cultivar ECW‐10R. The results revealed that the Δ*vemR* mutant induced a delayed and weakened HR compared to the wild‐type strain. As shown in Figure 2b, the Δ*vemR* mutant did not induce any visual HR symptoms until 16 h postinoculation (hpi), while the wild‐type strain triggered typical HR symptoms. Moreover, the complemented strain CΔ*vemR* (the mutant Δ*vemR* harbours an entire *vemR* coding sequence cloned into the vector pLAFR3; Table S1) could stimulate wild‐type HR symptoms. The effect of VemR mutation on HR induction was further substantiated using the electrolyte leakage assay. Here, leaf tissues within the infiltration areas were collected at four time points (0, 8, 16, and 24 hpi). The results showed that the wild‐type strain 8004 and the complemented strain CΔ*vemR* induced similar levels of electrolyte leakage, whereas the mutant Δ*vemR* induced much lower levels at 8, 16, and 24 hpi compared to the wild type at the same time points (Figure 2c). At 0 hpi, it appeared that all of the tested strains generated a similar electrolyte leakage value (Figure 2c), therefore these results indicate that VemR plays a positive role in full HR induction.

**FIGURE 3:**
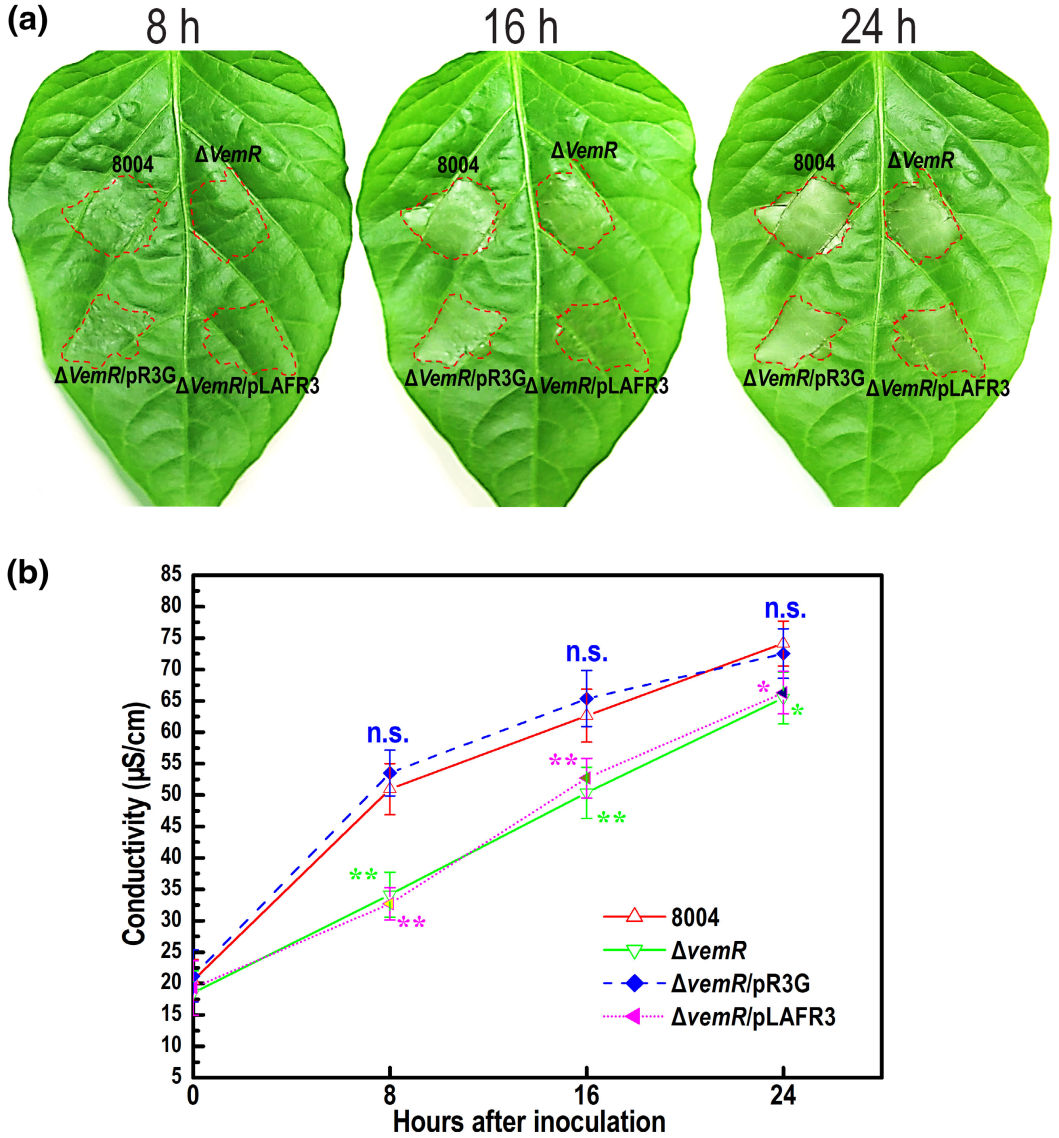
Constitutively expressing *hrpG* in the *vemR* mutant restores its ability of hypersensitive response (HR) induction in nonhost plant. The *Xanthomonas campestris* pv. *campestris* (Xcc) wild‐type strain 8004, *vemR* deletion mutant Δ*vemR*, cross‐complemented strain Δ*vemR*/pR3G (Δ*vemR* constitutively expressing *hrpG*), and Δ*vemR*/pLAFR3 (Δ*vemR* harbouring an empty vector pLAFR3) were cultured in nutrient‐yeast‐glycerol (NYG) medium overnight. Bacterial cells were collected, washed with, and resuspended in 10 mM sodium phosphate buffer to a density of 10^7^ cfu/ml. Approximately 5 μl of bacterial resuspension was infiltrated into the leaf mesophyll. (a) The HR symptoms recorded at 8, 16, and 24 h postinoculation (hpi). Three replications were performed in each experiment, and the experiment was repeated three times. The results presented are from a representative experiment, and similar results were obtained in all other independent experiments. (b) The electrolyte leakage from pepper leaves inoculated with Xcc strains. The conductivity of the infiltrating spots was measured at 0, 8, 16, and 24 hpi, with four 0.4 cm^2^ leaf disks collected from the infiltrated area for each sample. Three samples were taken for each measurement in each experiment. Data are shown as mean and standard deviation. Significance was determined by analysis of variance and Dunnett's post hoc test for comparison to the wild type. *p < 0.05, **p < 0.01; n.s., not significant. The experiment was repeated three times with similar results

To get a clue about the hierarchy of VemR and HrpG in the regulation of T3SS expression, we expressed constitutively *hrpG* in the *vemR*‐deleted mutant Δ*vemR* and determined its phenotypes. To achieve this, the entire open reading frame (ORF) sequence of the *hrpG* gene was cloned into the plasmid pLARF3 to generate the recombinant plasmid pR3G (Table S1), which was then introduced into the mutant strain Δ*vemR*. The obtained cross‐complemented strain, named Δ*vemR*/pR3G (Table S1), was tested for HR induction in the nonhost plant pepper ECW‐10R. The result showed that Δ*vemR*/pR3G induced a wild‐type HR symptom, while the control strain Δ*vemR*/pLAFR3 produced a delayed and weakened HR similar to that caused by the Δ*vemR* mutant (Figure 3a). The effect of constitutively expressing *hrpG* in Δ*vemR* on HR induction was further substantiated using the electrolyte leakage assay. As shown in Figure 3b, the electrolyte leakage values induced by Δ*vemR*/pR3G and the wild type were at a similar level at all of the tested time points. These combined data indicate that the HR‐eliciting capability of the Δ*vemR* mutant can be completely restored by constitutively expressing *hrpG*. Although it is unclear whether constitutively expressing HrpG in the *vemR* deletion mutant could completely recover the production of the effectors responding for HR induction, the above results suggest that the *vemR* mutant with constitutive expression of HrpG could produce the HR induction effector enough for a wild‐type HR stimulation.

**FIGURE 4:**
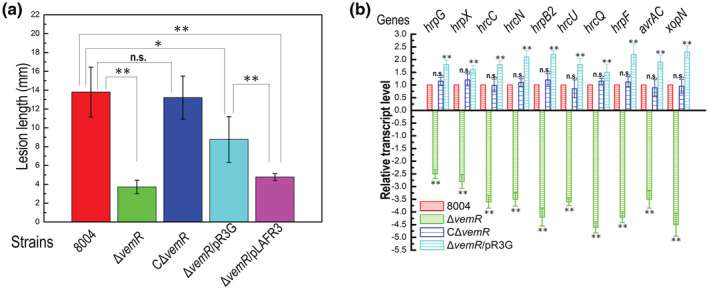
Constitutively expressing *hrpG* in the *vemR* mutant partially restores its virulence in host plant. (a) Virulence test of *Xanthomonas campestris* pv. *campestris* (Xcc) strains. The Xcc strains were cultured in nutrient‐yeast‐glycerol (NYG) medium overnight and bacterial cells were collected and resuspended to a concentration of 10^7^ cfu/ml (OD_600_ of 0.01). Chinese radish (*Raphanus sativus*) leaves were cut with scissors dipped in the bacterial suspensions. Lesion lengths (disease symptoms) were scored at 10 days postinoculation. Values given are the mean and *SD* from 15 inoculated leaves in one experiment. Analysis of variance (ANOVA) and Dunnett's post hoc test were used to identify significant differences which are indicated with asterisks. *p < 0.05, **p < 0.01; n.s., no significant. The experiment was repeated three times with similar results. (b) The expression levels of *hrp* genes in the Xcc wild‐type strain 8004, the *vemR* deletion mutant Δ*vemR*, and the cross‐complemented strain Δ*vemR*/pR3G in the host plant. Xcc strains were inoculated into Chinese radish leaves by infiltrating with a syringe. The infiltrated leaf part was collected 24 h after inoculation, and total RNA was extracted and reverse transcription‐quantitative PCR was performed. Values given are the mean ± *SD* of triplicate measurements from a representative experiment. Differences were determined by ANOVA and Dunnett's post hoc test. **p < 0.01; n.s., not significant. Similar results were obtained in two other independent experiments

The virulence of the mutant strain Δ*vemR*/pR3G was also determined by the leaf clipping inoculation method in the host plant Chinese radish. Ten days after inoculation, Δ*vemR*/pR3G caused disease symptoms with a mean lesion length of 8.7 mm, which was significantly more severe than that caused by the mutant Δ*vemR* (mean lesion length 3.2 mm) but significantly less severe compared to the wild‐type strain (mean lesion length 13.8 mm) and the complemented strain CΔ*vemR* (mean lesion length 13.2 mm) (Figure 4a). This indicates that constitutive expression of *hrpG* could only partially restore the virulence of the VemR‐deficient mutant.

In parallel, whether constitutively expressing *hrpG* in the mutant Δ*vemR* restores the transcriptional level of the T3SS genes in planta was investigated by RT‐qPCR. The strains 8004, Δ*vemR*, CΔ*vemR*, and Δ*vemR*/pR3G were inoculated into Chinese radish leaves by infiltration. RNAs were extracted from the infected leaves at 24 hpi and subjected to RT‐qPCR analysis using specific primers designed for determination of some *hrp*/*hrc* (*hrcC*, *hrcN*, *hrcQ*, *hrcU*, *hrpF*, *hrpG*, and *hrpX*) and T3E genes (*avrAC* and *xopN*) (Table S3). As shown in Figure 4b, the mRNA levels of all the tested genes in Δ*vemR*/pR3G were fully recovered to the wild‐type level, even to a higher level than the wild type. As expected, the mRNA levels of the determined genes were obviously decreased in Δ*vemR* compared to the wild‐type strain and completely restored to the wild‐type level in CΔ*vemR* (Figure 4b). These results suggest that constitutive expression of *hrpG* in the mutant lacking *vemR* can restore the expression of *hrp* genes in planta. Taken together, the above combined data indicate that VemR positively regulates *hrp* genes during infection and controls virulence partially by regulating the expression of T3SS.

### 2.3 VemR physically interacts with a subset of proteins including HrpG

As mentioned above, previous works demonstrated that VemR physically interacts with FleQ, FliM, and RavA to control bacterial motility (Li et al., 2020; Lin et al., 2022). As deletion of VemR influences many other cellular processes in addition to cell motility, the scope of regulation by VemR in Xcc cannot be completely accounted for by FleQ, FliM, and RavA. To identify other proteins interacting with VemR, we employed co‐immunoprecipitation (Co‐IP) coupled with liquid chromatography tandem‐mass spectrometry (LC–MS/MS) as previously described (Li et al., 2022). To this end, we constructed the strain 8004/VemR::3 × FLAG, which chromosomally expresses VemR protein fused with a 3 × FLAG‐tag (VemR::3 × FLAG) (Table S1). A western blot assay confirmed that the VemR::3 × FLAG fusion protein could be eluted from the strain 8004/VemR::3 × FLAG, but not the wild‐type strain 8004 (Figure S1). Protein complexes with VemR::3 × FLAG in Xcc cells were purified and analysed by LC–MS/MS. This analysis identified a number of putative interacting proteins for VemR (Table S4). Among these proteins, 17 (XC_0638, XC_1060, XC_1186, XC_1187, XC_1358, XC_1359, XC_1625, XC_2284, XC_2243, XC_2245, XC_2266, XC_2267/FliM, XC_2268/FliN, XC_2282/CheY, XC_2284/CheA, XC_2304, and XC_2311) are involved in chemotaxis and motility, seven (XC_1061/PilR, XC_1261, XC_2229/RavA, XC_3067, XC_3670/HpaS, XC_3077/HrpG, and XC_4031) are TCS members, and five (XC_0984, XC_2723, XC_2767/Fur, XC_2801, and XC_3597) are transcriptional regulators or DNA‐binding proteins. Among the four previously identified VemR‐interacting proteins (i.e., FliM, FleQ, HpaS, and RavA), three (FliM, HpaS, and RavA) were discovered under our test conditions. The reason why FleQ was absent is unknown. Importantly, the T3SS key regulator HrpG was identified as a potential VemR‐interacting protein in the experiment.

**FIGURE 5:**
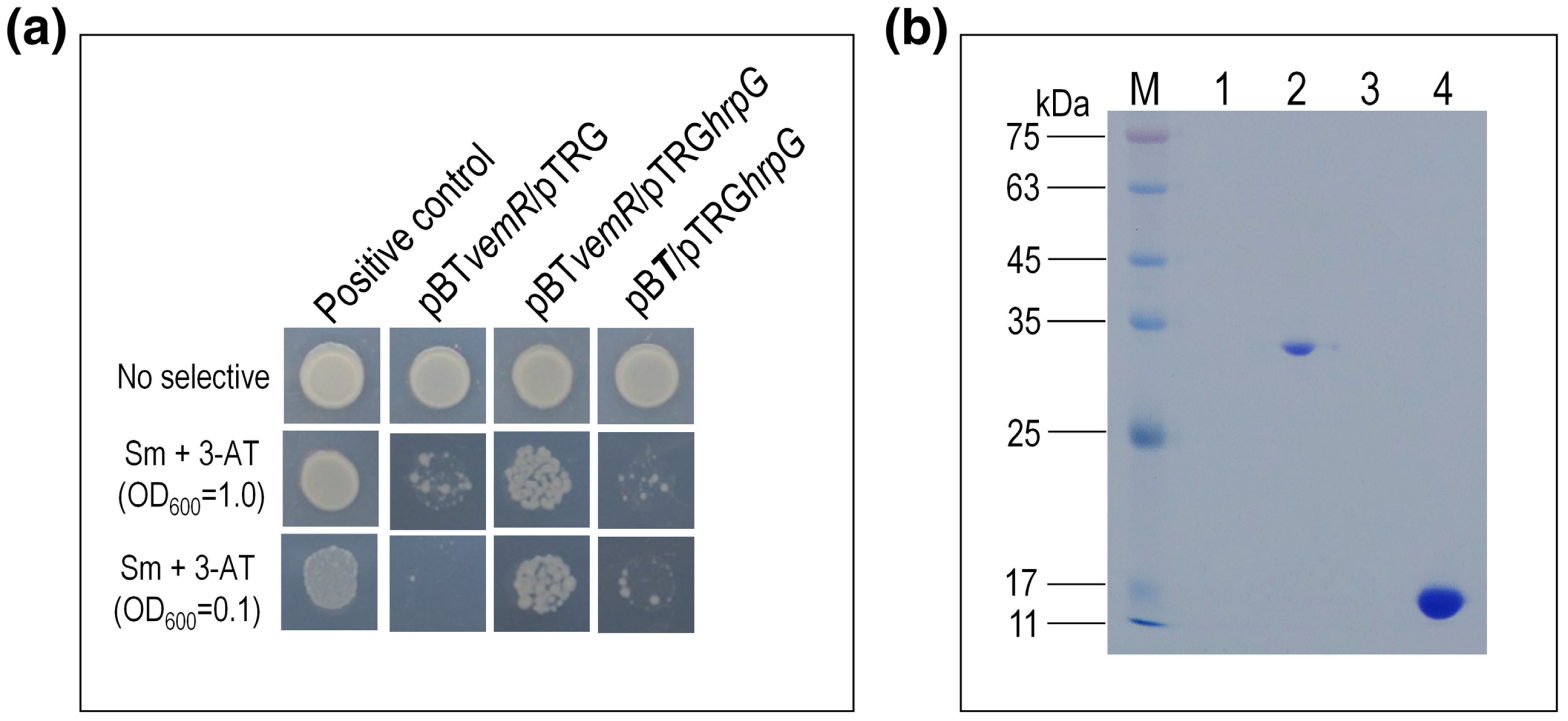
Physical interaction test between VemR and the type III secretion system (T3SS) key regulatory protein HrpG of *Xanthomonas campestris* pv. *campestris* (Xcc). (a) Bacterial two‐hybrid experiment. The reporter strain *Escherichia coli* XL1‐blue MRF′ with different plasmid pairs was grown on nonselective plates (inoculated with a cell concentration of OD_600_ = 1.0) and double‐selection indicator plates (inoculated with cell concentrations of OD_600_ = 1.0 and 0.1) containing 3‐amino‐1,2,4‐triazole (3‐AT) and streptomycin (Sm). Protein–protein interactions activate the expression of *addA* and *HIS3* genes within the reporter gene cassette of the reporter strain, resulting in resistance to 3‐AT and Sm. The reporter strain with the plasmid pair pBTPrtA‐pTRGMip was used as a positive control. Three independent experiments showed similar results. (b) Pull‐down assay. 6 × His‐tagged HrpG protein and other 6 × His‐tagged proteins were overexpressed and purified. Bait protein HrpG was biotinylated and 50 μl of 0.6 μg/μl biotinylated HrpG protein was immobilized to streptavidin Sepharose beads. The potential prey protein VemR was mixed with the bait protein and incubated. The HupB and McvR proteins were used as positive and negative controls, respectively, in addition to the negative control that 6 × His::VemR was mixed with streptavidin Sepharose beads. After elution, samples were separated on 12% SDS‐PAGE and visualized by Coomassie blue staining. Lane 1, 6 × His::VemR was mixed with streptavidin Sepharose beads; lane 2, pull‐down of 6 × His::HupB by biotinylated HrpG; lane 3, biotinylated HrpG was mixed with 6 × His::McvR; lane 4, pull‐down of 6 × His::VemR by biotinylated HrpG; M, molecular mass marker. Three independent experiments showed the same result

Given that VemR affects the expression of T3SS genes, we further validated its interaction with the T3SS key regulator HrpG using the bacterial two‐hybrid (B2H) assay as previously described (Li et al., 2020). To do this, *vemR* and *hrpG* were amplified by PCR using the corresponding primer sets (Table S3) and cloned into the bait vector pBT and the prey vector pTRG, respectively, resulting in the recombinant plasmids pBT*vemR* and pTRG*hrpG* (Table S1). The obtained recombinant plasmids were cotransformed into the reporter *Escherichia coli* XL1‐Blue MRF′ (Table S1), and the resulting strain was tested on double‐selective indicator plates containing 3‐amino‐1,2,4‐triazole and streptomycin. As shown in Figure 5a, similar to the positive control strain expressing Mip and PrtA proteins which have been shown to interact with each other (Meng et al., 2011), the reporter strain expressing VemR and HrpG could grow on the selective plates. However, the strains expressing either HrpG or VemR could not grow (Figure 5a). These data suggest that HrpG and VemR can physically interact with each other. This was further substantiated by a pull‐down biotinylated protein–protein assay. To do this, recombinant 6 × His‐tagged proteins HrpG and VemR were overproduced. After purification, the recombinant proteins were subjected to the pull‐down assay. The 6 × His‐tagged HupB, which has been shown to interact with HrpG (Zhang et al., 2020), and the 6 × His‐tagged McvR (Li et al., 2022) were used as a positive and a negative control, respectively. As shown in Figure 5b, HrpG could capture HupB (lane 1) as well as VemR (lane 2) but not McvR (lane 3), supporting that VemR interacts physically with HrpG.

Taken together, the above data from the Co‐IP coupled with LC–MS/MS, B2H, and pull‐down assays indicated that a physical interaction exists between the single‐domain response regulator VemR and the T3SS key regulator HrpG.

### 2.4 VemR enhances the activation of *hrpX* transcription by HrpG in vitro

**FIGURE 6:**
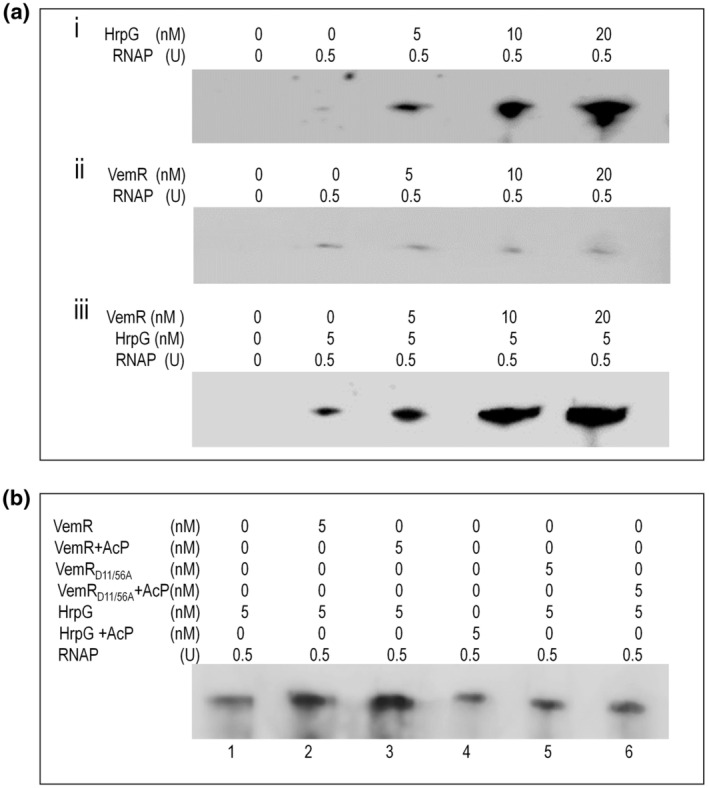
VemR enhances *hrpX* transcription activated by HrpG in vitro. RNA was generated from a 311‐bp template DNA fragment extending from −131 to +179 relative to the transcriptional initiation site (TIS) of the *hrpX* promoter using *Escherichia coli* RNA polymerase (RNAP) holoenzyme. Transcription products were run on a 5% denatured polyacrylamide gel containing 7 M urea in Tris‐borate‐EDTA electrophoresis buffer. (a) VemR enhances the activation of HrpG on the transcription of *hrpX*. Template DNA was incubated with various amounts of HrpG (i), VemR (ii) or HrpG with various amounts of VemR protein (iii), 0.5 U of RNAP was added prior to the initiation of the reactions. Transcription products (2 μl) were then run on the gel. The amounts of RNAP, VemR, and HrpG used are indicated at the top. All experiments were replicated more than three times with similar results and representative results are shown. (b) Phosphorylation of VemR is important for its function. Reactions were carried out with DNA fragments of the *hrpX* and series of proteins. The amounts of proteins used are indicated above the photographs. The experiment was repeated more than three times and similar results were obtained

The fact that VemR physically interacts with HrpG implies that VemR may affect the regulatory efficiency of HrpG in Xcc. To validate this possibility, we determined whether VemR affects the transcriptional level of *hrpX* gene activated by HrpG in vitro. Previous studies showed that *hrpX* is a direct target of HrpG (Ficarra et al., 2015; Zhang et al., 2020). In this study, we performed several in vitro transcription assays using a 311‐bp template DNA fragment containing the *hrpX* promoter of the Xcc wild‐type strain 8004 and the RNA polymerase (RNAP) holoenzyme from *E. coli*. In the experiments, we first tested the influence of different amounts of purified HrpG or VemR on *hrpX* transcriptional level. As shown in Figure 6a, when 0.5 U RNAP was used, a certain amount of *hrpX* transcripts could be generated without addition of HrpG or VemR protein. The *hrpX* transcript level was significantly increased when HrpG protein was added to the reaction and the transcript level increased along with the addition of increasing amount of HrpG (from 5 to 20 nM) (Figure 6a‐i). However, there was no detectable variety in the *hrpX* transcript level along with the increase of VemR (from 5 to 20 nM) (Figure 6a‐ii), suggesting that unlike HrpG, VemR itself does not directly regulate *hrpX*. Given that VemR does not harbour a DNA‐binding motif and can physically interact with HrpG, it is probable that VemR can enhance the efficiency of HrpG activating *hrp*X transcription by an unknown mechanism. To verify this probability, an in vitro transcription assay was performed by adding varied amounts (from 5 to 20 nM) of VemR in the reactions containing the same concentration (5 nM) of HrpG. The results showed that addition of VemR enhanced obviously the transcript level of *hrpX* and the enhancement was more conspicuous when the amount of VemR was increased from 5 to 20 nM (Figure 6a‐iii), implying that VemR indeed acts as an enhancer for *hrpX* transcription activated by HrpG.

### 2.5 The phosphorylation of VemR is crucial for its function in enhancing *hrpX* expression

It is known that in VemR the aspartyl residues at positions 11 and 56 are required for phosphorylation and the phosphorylation is essential for VemR to regulate cell motility and EPS production (Li et al., 2020; Tao & He, 2010). Here, we determined whether the aspartyl residues are also important for VemR to modulate *hrpX* expression. A variant of VemR, named VemR_D11/56A_, was constructed by replacement of the aspartyl residues at positions 11 and 56 with alanine in VemR. Then, the effect of the variant together with HrpG on the transcription of *hrpX* was estimated by in vitro transcription assay. As shown in Figure 6b, the variant VemR_D11/56A_ plus HrpG (lane 5) produced a *hrpX* transcript level similar to HrpG alone (lane 1), which was significantly lower than that produced by VemR with HrpG (lane 2). Our previous work has revealed that VemR can be phosphorylated by the acetyl phosphate (AcP), which specifically phosphorylates the acceptor aspartyl residues of certain TCS RRs (Li et al., 2020). In the transcription assay, AcP was added in the reactions with the wild‐type VemR or the variant VemR_D11/56A_. As shown in Figure 6b, addition of AcP in VemR (lane 3) could visibly improve *hrpX* transcript level compared to VemR (lane 2), while the *hrpX* transcript levels were similar in the reactions of VemR_D11/56A_ with and without AcP (lanes 5 and 6).

In addition, no transcript was observed when a 121‐bp internal fragment of the *hrpX* gene (from +1 to +121 relative to the transcriptional initiation site) was used as the template instead of the 311‐bp fragment containing *hrpX* promoter (data no shown). Taken together, these data suggest that the phosphorylation at the aspartyl residues at positions 11 and 56 of VemR is probably important for its regulatory function in the enhancement of *hrpX* transcription by HrpG.

### 2.6 VemR acts as an enhancer for T3Es production

**FIGURE 7:**
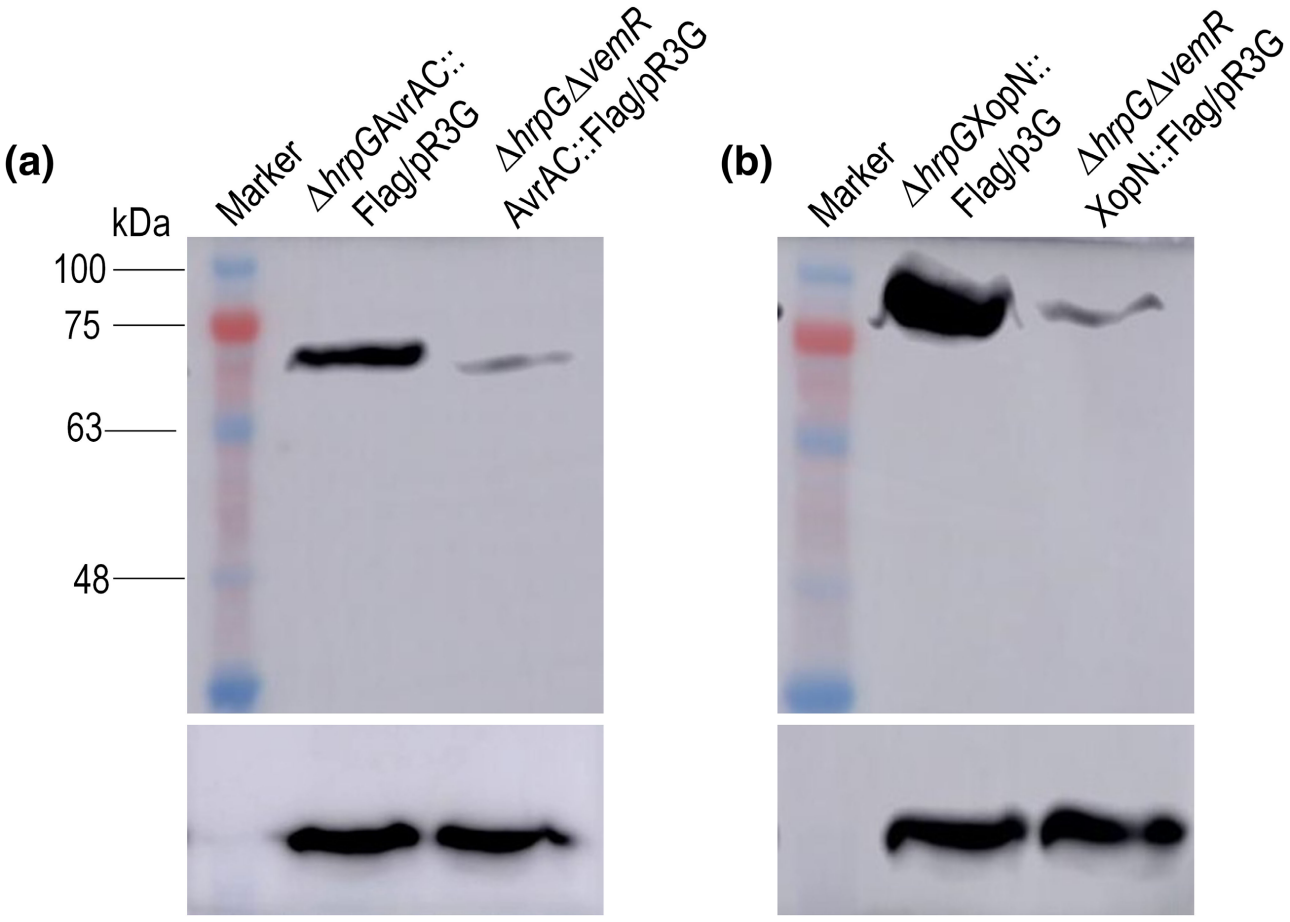
Western blot assays revealed that mutation in VemR reduces the level of type III effectors (T3Es) production. Strains ∆*hrpG*AvrAC::FLAG/pR3G, ∆*hrpG*∆*vemR*AvrAC::FLAG/pR3G, ∆*hrpG*XopN::FLAG/pR3G, and ∆*hrpG*∆*vemR*XopN::FLAG/pR3G were cultured in XVM2 medium for 12 h and total proteins were prepared. Thirty micrograms of protein for each strain was electrophoresed in SDS‐PAGE and transferred to a PVDF membrane. The presence of AvrAC protein in strains ∆*hrpG*AvrAC::FLAG/pR3G and ∆*hrpG*∆*vemR*AvrAC::FLAG/pR3G (a) or XopN protein in strains ∆*hrpG*XopN::FLAG/pR3G and ∆*hrpG*∆*vemR*XopN::FLAG/pR3G (b) was detected by anti‐FLAG‐tag mouse monoclonal antibody. As a loading reference, the blot was also probed with an anti‐RNA polymerase β‐antibody (low element). Experiments were replicated three times with similar results and representative results are shown

Our in vitro transcription results reveal that VemR can enhance the activation of *hrpX* by HrpG. As described above, HrpX is a crucial regulator for T3SS. In the study, we further validated the function of VemR by measurement of the T3Es produced by the *vemR* deletion mutant. It is known that in addition to activating the transcription of *hrpX*, HrpG can also bind to its own promoter to repress its own expression (Ficarra et al., 2015; Wengelnik et al., 1996). The transcriptome and RT‐qPCR analyses showed that mutation of *vemR* significantly affected the expression of *hrpG*. To gain a better insight into the regulatory mechanism of VemR, we detected the T3Es production using a *vemR* deletion mutant with constitutive expression of *hrpG* to exclude the potential influence of *hrpG* self‐regulation. To achieve this, we constructed a *hrpG* deletion mutant, named ∆*hrpG* (Table S1), and a *hrpG* as well as *vemR* double‐deletion mutant, named ∆*hrpG*∆*vemR* (Table S1). The mutant strains were further modified by fusing a 3 × FLAG‐tag to the T3E AvrAC or XopN (AvrAC::3 × FLAG or XopN::3 × FLAG) by in‐frame insertion of the 3 × FLAG‐coding sequence into the 3′ end of the AvrAC‐ or XopN‐coding sequence in the genome of ∆*hrpG* and ∆*hrpG*∆*vemR*, generating strains named ∆*hrpG*AvrAC::3 × FLAG and ∆*hrpG*∆*vemR*AvrAC::3 × FLAG, or ∆*hrpG*XopN::3 × FLAG and ∆*hrpG*∆*vemR*XopN::3 × FLAG (Table S1), respectively. We chose AvrAC and XopN because the expression of their coding genes is positively regulated by HrpX (Jiang et al., 2008; Xu et al., 2008). In Xcc, the effector responding for HR induction in the pepper plant used in this study is AvrBs1 (Xu et al., 2008). However, as Xcc produces a very small amount of AvrBs1 protein in media, we did not include AvrBs1 detection in the experiment. To constitutively express *hrpG*, the recombinant plasmid pR3G (Table S1) was introduced into these strains by triparental conjugation. The resulting transconjugant strains, named ∆*hrpG*AvrAC::FLAG/pR3G, ∆*hrpG*∆*vemR*AvrAC::FLAG/pR3G, ∆*hrpG*XopN::FLAG/pR3G, and ∆*hrpG*∆*vemR*XopN::FLAG/pR3G (Table S1), were used to test the AvrAC protein or XopN protein level by western blot analysis. As shown in Figure 7, the AvrAC protein level in the wild‐type VemR strain ∆*hrpG*AvrAC::FLAG/pR3G was much higher than that in the VemR‐deficient strain ∆*hrpG*∆*vemR*AvrAC::FLAG/pR3G. Similarly, the level of XopN in the wild‐type VemR strain ∆*hrpG*XopN::FLAG/pR3G was obviously higher compared to the VemR‐deficient strain ∆*hrpG*∆*vemR*XopN::FLAG/pR3G. These data suggest that VemR acts as an enhancer for T3Es production.

## 3 DISCUSSION

Previous studies have shown that the SD‐RR VemR is crucial for the virulence, cell motility, and production of EPS and exoenzymes of Xcc (Li et al., 2020; Tao & He, 2010). In the present study, we uncovered by transcriptome analysis that VemR is a global regulator modulating the expression of more than 10% of the annotated protein‐encoding genes in Xcc. Phylogenetic classification reveals that, in addition to the previously known phenotypes, many other cellular processes including the essential pathogenicity determinant T3SS are also under the control of VemR. VemR is a highly conserved protein in all *Xanthomonas* species and it was reported that mutation of the *vemR* in the citrus canker pathogen *Xanthomonas citri* subsp. *citri* and the rice bacterial leaf streak agent *Xanthomonas oryzae* pv. *oryzicola* also resulted in a reduction of EPS, motility, virulence, and HR (Cai et al., 2022; Wu et al., 2019). It is possible that the VemR proteins among *Xanthomonas* species have similar functions.

Our data showed that deletion of VemR led to an increase in *hrpX* expression in the rich medium NYG but a decrease in *hrpX* expression in the minimal medium XVM2 and in planta, indicating that VemR regulates *hrpX* expression negatively in nutrient‐rich conditions but positively in nutrient‐deficient conditions. Notably, in vitro transcription assay demonstrated that phosphorylated VemR significantly enhanced the activation of *hrpX* transcription by HrpG, which is consistent with VemR positively regulating *hrpX* expression in nutrient‐deficient condition but inconsistent with VemR negatively regulating *hrpX* expression in nutrient‐rich conditions. As previously reviewed (Mole et al., 2007; Tang et al., 2006), it is well established that in xanthomonads the expression of the *hrp* genes is repressed in rich media but induced in planta or in certain minimal media. However, the regulatory mechanism controlling such an expression manner is currently unknown. To date, a number of regulators involved in *hrp* gene induction, such as HpaS, HrpG, and HrpX, have been identified and characterized, providing some insights into the complex regulation mechanism of *hrp* gene activation (Alvarez‐Martinez et al., 2020; An et al., 2020; Mole et al., 2007). However, only a few regulators have been reported to repress *Xanthomonas hrp* gene expression and their regulatory mechanism remains to be explored (An et al., 2011; Lu et al., 2011). Similar to VemR, HpaR1, a GntR family transcriptional regulator in Xcc, was previously demonstrated to influence *hrp* gene expression with opposite regulations in response to different environments (An et al., 2011). HpaR1 regulates *hrp* gene expression via *hrpG* negatively in standard media but positively in plant tissues (An et al., 2011). Recently, Zhang et al. (2020) found by ChIP‐seq that in Xcc HrpG could bind to the *vemR* promoter region in vivo and Cai et al. (2022) showed that in *X. oryzae* pv. *oryzicola* the transcription of *vemR* is repressed by HrpG in nutrient‐rich conditions. Taken together, these data suggest that VemR and HrpG play important roles in both induction and repression of *hrp* gene expression in different growth conditions. This work focused on the function of VemR in positive regulation of *hrp* genes. The mechanism by which VemR represses *hrp* gene expression in nutrient‐rich conditions needs to be further investigated.

Our Co‐IP coupled with LC–MS/MS, B2H and pull‐down assays confirm that VemR physically interacts with HrpG (Figure 5). Furthermore, the *vemR* deletion mutant is deficient in full HR induction and constitutive expression of *hrpG* restores full HR to the mutant (Figures 2 and 3). These combined data suggest that the influence of VemR on HR induction is probably through HrpG. As described above, HrpG is an OmpR family DNA‐binding regulator that directly binds to the promoter region of *hrpX* to activate its transcription. In this study an in vitro transcription experiment was conducted to validate the suggestion. The results showed that HrpG indeed could activate the transcription of *hrpX* in vitro. More importantly, the experiment showed clearly that VemR alone did not alter the *hrpX* transcript level, but it could obviously increase the *hrpX* transcripts initiated by HrpG. These results support the finding that VemR acts as an enhancer to improve the transcriptional activation of *hrpX* by HrpG. Notably, the *hrpX* transcript level could be significantly increased along with raising the HrpG concentration from 5 to 20 nM, without addition of VemR. In addition, the *hrpX* transcript level generated in the reaction with 5 nM HrpG and 10 nM VemR was similar to that generated in the reaction with 20 nM HrpG only. These data may explain why constitutively expressing *hrpG* could restore full HR of the VemR‐deficient mutant. As expected, constitutive expression of *hrpG* could only partially return virulence to the VemR‐deficient mutant (Figure 4a) because that in addition to the T3SS regulated by HrpG/HrpX, VemR also plays an important role in cell motility and the production of EPS and exoenzymes, which are not regulated by HrpG/HrpX but important for Xcc virulence.

Currently, we do not know the detailed mechanism by which VemR improves the function of HrpG. The in vitro transcription assay demonstrated that in the reaction with HrpG plus VemR, the *hrpX* transcripts were significantly increased after addition of AcP, and that addition of AcP to the reaction with HrpG or HrpG plus VemR_D11/56A_ did not alter the transcriptional level of *hrpX*. In addition, our previous work has confirmed that VemR can be phosphorylated by AcP in vitro and its aspartyl residues at positions 11 and 56 are essential for the phosphorylation (Li et al., 2020). These combined results suggest that AcP maybe improves the phosphorylation of VemR, thereby enhancing the activation of *hrpX* by HrpG, but cannot improve the activity of HrpG. It is possible that AcP has no effect on HrpG phosphorylation under the test conditions. Based on these results, we assume that a possible regulatory mechanism for VemR in *hrpX* expression may be that the binding of phosphorylated VemR to HrpG could modulate the activation activity of HrpG to *hrpX* promoter, thereby increasing the transcription level of *hrpX*. Thus, VemR acts as an accessory element to HrpG protein involved in controlling T3SS in Xcc. Notably, there are several TCS members and non‐TCS member DNA‐binding proteins that might physically interact with VemR, revealed by the Co‐IP coupled with LC–MS/MS assay, implying that the phosphorylated VemR may interact with these regulators in multilayered control of diverse cellular processes. To further verify whether VemR binds to these proteins to alter their impact on target gene expression is of great value to broaden our understanding of the function of VemR. Alternatively, given that HrpG and HpaS compose a TCS, VemR may act as an intermediate in the phosphorelay between HrpG and its cognate sensor kinase HpaS to control *hrpX* expression in vivo. It is also possible that binding of VemR to HrpG can increase the stability of HrpG protein or modulate the binding strength of HrpG to *hrpX* promoter, thereby increasing the transcription of *hrpX*. Further works are required to test these hypotheses.

**FIGURE 8:**
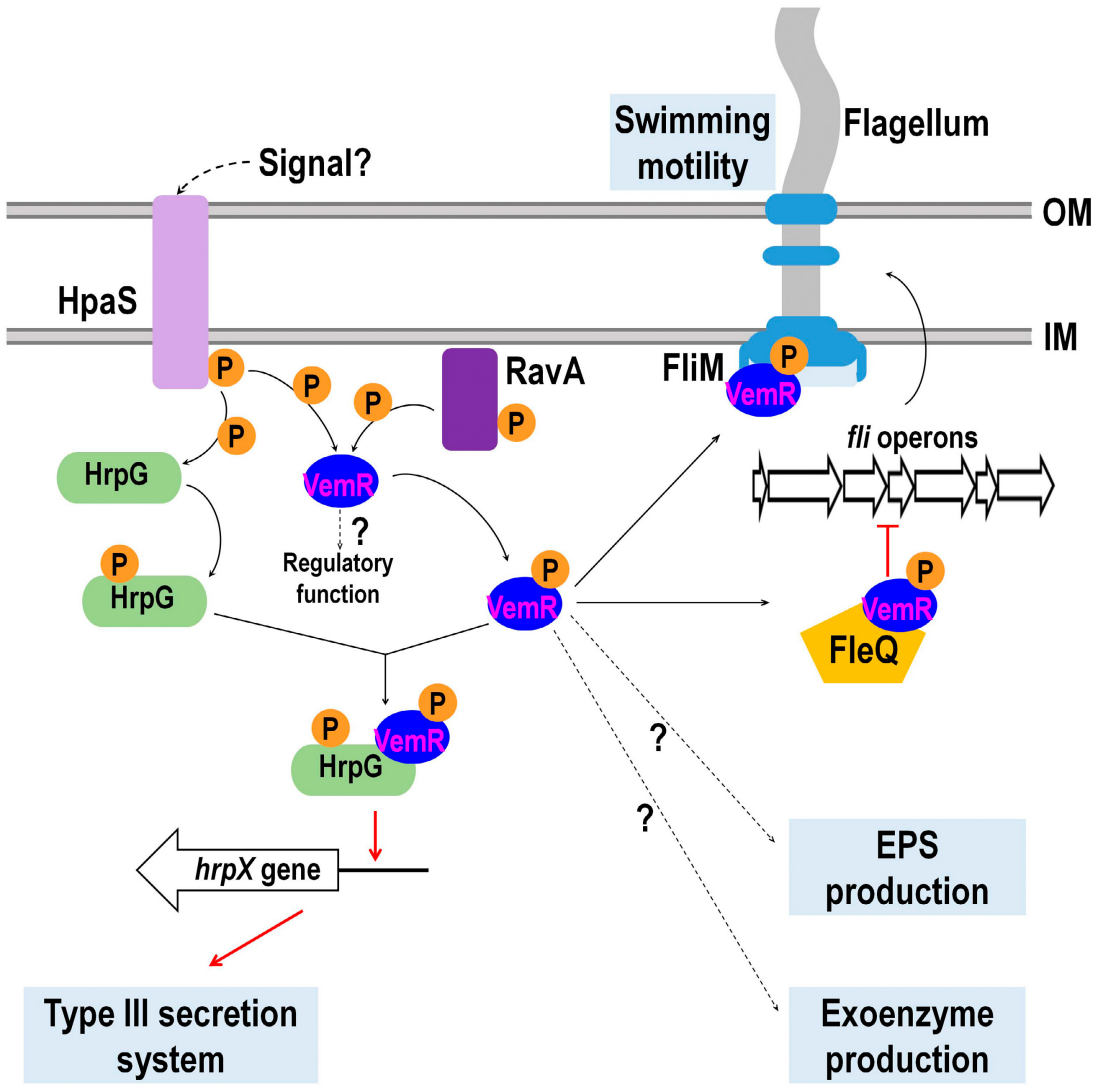
A model indicating the roles of the single domain response regulator VemR in *Xanthomonas campestris* pv. *campestris* (Xcc). As it does not have an output domain, VemR is supposed to recruit certain proteins to execute its regulatory functions. This study demonstrates that phosphorylated VemR regulates the type III secretion system (T3SS) by directly interacting with the two‐component signalling system (TCS) response regulator HrpG and enhancing its activation activity on *hrpX* expression. The TCS HK sensor HpaS can phosphorylate HrpG and VemR. In addition to HpaS, the TCS HK sensor RavA can also phosphorylate VemR. Moreover, in addition to interacting with FliM to modulate cell swimming motility, phosphorylated VemR can also interact with the transcriptional activator FleQ to repress the expression of the flagellar genes. However, how VemR regulates extracellular polysaccharide (EPS) and exoenzyme production is unclear. Whether the unphosphorylated VemR playing a regulatory role is unknown. Red arrow, positive regulation; red line with endbar, negative regulation

As mentioned above, previous studies have shown that HpaS and HrpG form a TCS that regulates the expression of *hrpX* (Li et al., 2014), and that HpaS can also form another TCS with VemR to control EPS production and cell motility (Li et al., 2020). Notably, constitutively expressing VemR in the *hpaS* deletion mutant could partially restore HR induction and T3SS expression (Figure S2). Furthermore, deletion of both *hpaS* and *vemR* caused a more severe reduction in T3SS expression and HR induction than deletion of *hpaS* or *vemR* only (Figure S2). The data obtained in this study combined with the previous results suggest that HpaS can modulate directly HrpG activity and indirectly via VemR. As HpaS is a membrane‐bound sensor kinase, it might play a distinct role in sensing environmental stimuli and transducing signals to regulate T3SS expression. In addition, it has been recently reported that the TCS sensor RavA can also interact with and phosphorylate VemR, and that VemR can interact with the transcriptional activator FleQ to repress the transcription of flagellum‐related genes (Lin et al., 2022). These findings indicate that VemR may function as an intermediate in signal transduction pathways involved in multiple physiological processes (Figure 8).

In conclusion, this work shows that the regulon of the TCS SD‐RR member VemR comprises a large portion of Xcc genes involved in many cellular processes. In addition to the previously known important virulence factors such as EPS and motility, VemR also controls the T3SS, a critical pathogenicity determinant. Taken together with previous studies, our results suggest that the phosphorylation of VemR might be crucial for its function and that VemR enhances the activation of *hrpX* by HrpG, probably via its interaction with HrpG (Figure 8). To the best of our knowledge, this is the first report that a SD‐RR very likely executes its function by directly interacting with a typical TCS response regulator with DNA‐binding ability to regulate gene expression. Although further studies are required to explore in depth the detailed functional mechanism of VemR, our findings provide some insights into the complex regulatory cascade of the HpaS/RavA‐VemR/HrpG‐HrpX signal transduction system in the control of T3SS expression. More importantly, our work suggests VemR probably plays a vital role in the signalling/regulatory network controlling virulence (Figure 8).

## 4 EXPERIMENTAL PROCEDURES

### 4.1 Bacterial strains, plasmids, and growth conditions

The bacterial strains and plasmids used in this study are listed in Table S1. *E. coli* strains were grown in Luria–Bertani medium (10 g tryptone, 10 g NaCl, 5 g yeast extract per litre) or M9 (67.8 g Na_2_HPO_4_, 30 g KH_2_PO_4_, 5 g NaCl, 10 g NH_4_Cl per litre) at 37°С. Xcc strains were grown at 28°С in NYG medium (Daniels et al., 1984), NY medium (NYG medium but without glycerol), and the minimal medium XVM2 (Astua‐Monge et al., 2005; Wengelnik & Bonas, 1996). Antibiotics were used as described previously (Li et al., 2020).

### 4.2 DNA and RNA manipulations

DNA manipulations followed the procedures previously described (Sambrook et al., 1989). Conjugations between Xcc and *E. coli* strains were performed as previously described (Turner et al., 1985). The restriction endonucleases T4 DNA ligase and *Pfu* DNA polymerase were provided by Promega. Total RNA extraction from bacterial culture or infected plant samples was carried out using RNAiso Plus Kit (TaKaRa) and cDNA was generated using a cDNA synthesis kit (Invitrogen). For measuring the transcription level of certain genes, RT‐qPCR was carried out as previously described (Li et al., 2022) using ChamQ universal SYBR qPCR master mix (Vazyme) with corresponding primers (Table S3) in a real‐time PCR thermal cycler (Analytik jena qTOWER2.0; Jena). The relative mRNA level was calculated with respect to the level of the corresponding transcript in the wild‐type strain 8004 (equalling 1). The expression level of the 16S rRNA gene was used as an internal standard. The RT‐qPCR tests were performed in triplicate.

### 4.3 Deletion mutant construction and complementation

*vemR* (*XC_2252*) single‐deletion mutant Δ*vemR* and the complemented strain CΔ*vemR* have been constructed in previous work (Li et al., 2020). To construct the *hrpG* (*XC_3077*) deletion mutant (or *hrpG* and *vemR* double deletion mutant), 724‐bp upstream and 698‐bp downstream fragments of the *hrpG* coding region were amplified using the corresponding primers (Table S3). The two fragments were cloned together into the vector pK18*mobsacB* (Schäfer et al., 1994). The resulting recombinant plasmid pK18*mobsacBhrpG* was introduced into Xcc 8004 (or Δ*vemR* mutant) by triparental conjugation. The obtained mutant was named Δ*hrpG* (or Δ*hrpG*Δ*vemR*) (Table S1). For cross‐complementation of the Δ*vemR* mutant, the recombinant plasmids pR3G (Table S1), which derived from an 899‐bp fragment containing promoterless *hrpG* gene cloned into the vector pLAFR3 (under the control of the *lacZ* promoter which expresses constitutively in Xcc), was transferred into the Δ*vemR* mutant by triparental conjugation, resulting in strain Δ*vemR*/pR3G.

### 4.4 Construction of the strains chromosomally encoding 3 × FLAG fused form protein

Xcc strains expressing the proteins fused with a 3 × FLAG‐tag at the C‐terminus were constructed using the method previously described (Li et al., 2022). Briefly, to construct a strain chromosomally encoding VemR::3 × FLAG for Co‐IP assays, a 428‐bp DNA fragment, which was composed of a 381‐bp VemR‐coding sequence and a 47‐bp FLAG‐coding sequence, was generated by PCR amplification using the genomic DNA of strain 8004 as template and the primer set LvemR‐FlagF/R (Table S3). Simultaneously, a 415‐bp DNA fragment, which was composed of a 39‐bp FLAG‐coding sequence, the 3‐bp stop codon of *vemR*, and 373‐bp downstream of the *vemR* stop codon, was generated by PCR amplification using the primer set RvemR‐FlagF/R (Table S3). The two fragments were joined using overlap extension PCR, and the resulting recombinant fragment was cloned into the suicide plasmid pK18*mobsacB* (Schäfer et al., 1994). The resulting recombined plasmid named pK*vemR*::*flag* (Table S1) was introduced into Xcc strain 8004 by conjugation, and the transconjugants chromosomally encoding VemR::3 × FLAG protein were screened and confirmed by the procedure described previously (Liu et al., 2019). The obtained variant strain was named 8004/VemR::3 × FLAG (Table S1). Similarly, to construct a strain chromosomally encoding AvrAC::3 × FLAG or XopN::3 × FLAG for western blotting, a recombinant fragment encoding an in‐frame 3 × FLAG peptide at the C‐terminus of AvrAC or XopN was obtained by PCR amplification with the corresponding primer sets (LavrAC‐FlagF/R and RavrAC‐FlagF/R for AvrAC::3 × FLAG, and LxopN‐FlagF/R and RxopN‐FlagF/R for XopN::3 × FLAG; Table S3). These obtained fragments were cloned into the suicide vector pK18*mobsacB*, and the resulting recombinant plasmids, named pK*avrAC*::*flag* and pK*xopN*::*flag* (Table S1), were introduced into the Xcc *hrpG* deletion mutant ∆*hrpG* and the *hrpG*/*vemR* double deletion mutant ∆*hrpG*∆*vemR*, respectively. The resulting strains were named ∆*hrpG*AvrAC::3 × FLAG, ∆*hrpG*∆*vemR*AvrAC::3 × FLAG, ∆*hrpG*XopN::3 × FLAG, and ∆*hrpG*∆*vemR*XopN::3 × FLAG (Table S1).

### 4.5 Co‐immunoprecipitation and LC–MS/MS analysis

Co‐IP combined with LC–MS/MS analysis was employed to identify the VemR‐interacting partners as previously described (Li et al., 2022). The strain 8004/VemR::3 × FLAG as well as the wild‐type strain 8004 (used as a negative control) were cultured in XVM2 medium overnight, and cells were collected and lysed. After centrifugation, the supernatant fractions were collected, and agarose‐conjugated anti‐FLAG was added and co‐incubated. The eluted proteins from agarose beads were resolved by SDS‐PAGE and analysed by LC–MS/MS on a nano‐LC system (Easy nLC 1000; Thermo Fisher Scientific) combined with an LTQ‐Orbitrap Elite mass spectrometer. The MS data were analysed with SEQUEST against Xcc 8004 Uniprot protein database (https://www.uniprot.org/taxonomy/314565). Peptides that were filtered with a confidence level of 99% were accepted. The FDR for peptide was set to 1%.

### 4.6 Western blotting

Western blot assays were performed followed the procedure described by Sambrook et al. (1989). Bacterial proteins separated by SDS‐PAGE were electrotransferred onto a polyvinylidene difluoride (PVDF) membrane (Millipore). After blocking with 1% milk, the proteins in the membrane were incubated with the 1:2500 diluted mouse anti‐DYKDDDDK‐tag mAb (Abmart) as the primary antibody, followed by washing four times with Tris‐buffered saline with Tween buffer (Tris 20 mM, NaCl 0.3 M, Tween 20 0.08% [vol/vol]). The diluted 1:5000 horseradish peroxidase (HRP)‐conjugated goat anti‐mouse IgG H&L (Beyotime Biotechnology) was used as the secondary antibody. After the membrane was washed for four times, the luminescence signal was detected according to the manufacturer's instructions. For a loading control, proteins were probed with the anti‐RNAP β‐antibody (EPR18704; Abcam) at 1:2000 dilution as the primary antibody, and the HRP‐conjugated goat anti‐rabbit IgG H&L as the secondary antibody.

### 4.7 Bacterial two‐hybrid assay

The BacterioMatch II two‐hybrid system (Stratagene) was carried out to detect the VemR–HrpG interaction as previously described (Li et al., 2014). Briefly, the 381‐bp *vemR* gene, obtained by PCR using the primer set vemR‐BTF/R (Table S3), was cloned into the bait vector pBT, generating the plasmid pBT*vemR* (Table S1). The 789‐bp *hrpG* coding sequence was PCR‐amplified from the Xcc strain 8004 with the primer set hrpG‐TRGF/R (Table S3) and cloned into the target vector pTRG, resulting the plasmid pTRG*hrpG* (Table S1). The plasmid pairs were cotransformed into the reporter strain *E. coli* XL1‐Blue MRF′. The cells of the resulting strains were resuspended in M9 medium (67.8 g Na_2_HPO_4_, 30 g KH_2_PO_4_, 5 g NaCl, 10 g NH_4_Cl per litre) and adjusted to a concentration of OD_600_ of 1.0 and 0.1. The bacterial suspensions were spotted on the nonselective plates and double‐selective indicator plates containing 5 mM 3‐amino‐1,2,4‐triazole and 12.5 μg/ml streptomycin, and then incubated at 28°С for 24 h.

### 4.8 Overproduction and purification of proteins

To overproduce HrpG protein, the 789‐bp *hrpG* coding sequence was PCR‐amplified from the Xcc strain 8004 using the primer set hrpG‐OF/R (Table S3). The obtained DNA fragment was cloned into the expression vector pGEX‐4T‐1. The resulting recombinant plasmid named pGEX‐HrpG (Table S1) was transformed into *E. coli* BL21. The obtained strain BL21/pGEX‐HrpG (Table S1) was cultured at 37°C for 1 h and induced by isopropyl β‐d‐thiogalactopyranoside (IPTG) at 25°C for 5 h, and then cells were harvested, lysed, and incubated with glutathione‐Sepharose beads at 4°C for 4 h. After three washes with phosphate‐buffered saline, glutathione‐S‐transferase (GST) fusion proteins bound to glutathione‐Sepharose beads were incubated at room temperature for 4 h with bovine thrombin (Solarbio). The proteins cleaved from the beads by the thrombin were used for in‐vitro transcription assays.

To obtain 6 × His‐tagged form of VemR and its variant VemR_D11/56A_, as well as HrpG, McvR and HupB proteins, *E. coli* strains M15/pQE‐30‐VemR, M15/pQE‐30‐VemR_D11/56A_, M15/pQE‐HrpG, BL21/pET‐30a‐McvR, and BL21/pET‐32a‐HupB expressing VemR, VemR_D11/56A_, McvR, and HupB, respectively, fused with a 6 × His‐tag on their N‐termini (Table S1) were grown and induced by IPTG. The cells were collected and the 6 × His‐tagged fusion proteins were purified using Ni‐NTA resin (Qiagen).

### 4.9 Protein pull‐down assay

Protein pull‐down assays were performed with the ProFound pull‐down biotinylated protein–protein interaction kit (Pierce) as previously described (Li et al., 2014). Briefly, the 6 × His::HrpG protein was biotinylated with sulfo‐NHS‐LC‐biotin. Then 50 μl of the purified biotinylated 6 × His::HrpG was incubated with streptavidin sepharose beads. After washing, beads were incubated with the samples containing the protein 6 × His::VemR, 6 × His::HupB or 6 × His::McvR. Then beads were washed and prey protein was eluted using elution buffer (pH 2.8). Twenty microlitres of the eluted sample were electrophoresed on 12% SDS‐PAGE and visualized by Coomassie brilliant blue staining.

### 4.10 In vitro transcription assays

In vitro transcription assays were performed as previously described (Su et al., 2016). Promoter sequence fragments (311 bp) of *hrpX* were acquired using PCR with the primer set hrpXivt‐F/R (Table S3). The obtained *hrpX* promoter sequence fragments and HrpG or/and VemR protein were incubated for 30 min at room temperature in transcription buffer. Then, a NTP mixture (250 μM each of ATP, CTP, and GTP, 250 μM biotin‐16‐UTP) and 0.5 U of *E. coli* RNA polymerase holoenzyme (New England BioLabs) were added to initiate transcription. After incubation at 28°C for 30 min, the reactions were terminated and the transcription products were analysed by electrophoresis. The transcripts obtained were visualized using a phosphor imager screen (GE AI600).

### 4.11 Transcriptome analysis

Transcriptome analysis was performed as previously described (Cui et al., 2018). The Xcc wild‐type strain 8004 and the *vemR* deletion mutant Δ*vemR* were cultured in NYG medium to an OD_600_ of 0.6, and RNA was then prepared. After the quantity determination and quality assessment, total RNA was sent to Novogene for library construction and strand‐specific RNA sequencing. Sequencing libraries were generated using a NEBNext Ultra Directional RNA Library Prep Kit for Illumina (New England BioLabs) and sequenced on an Illumina HiSeq 2000 platform. Clean reads were mapped to the reference genome and the RPKM (reads per kilobase per million mapped reads) method was used to calculate the gene expression levels. False discovery rate FDR ≤0.05 and |log_2_FC| (log_2_ of the fold changes) ≥1 were considered for differentially expressed genes (DEGs). For confirmation, several DEGs were selected randomly to perform RT‐qPCR analysis.

### 4.12 Plant assays

The virulence of Xcc strains to Chinese radish (*Raphanus sativus*) was tested by the leaf‐clipping method (Dow et al., 2003) with minor modification. Bacterial cells from overnight culture were collected, washed, and resuspended to a cell density of OD_600_ of 0.01 (approximately 10^7^ cfu/ml) in sterile ultrapure water. Leaves were cut with scissors dipped in the bacterial suspensions. Lesion length was measured 10 days after inoculation. Three replications were done in each experiment, and the experiment was repeated three times.

For RT‐qPCR analysis, leaves of Chinese radish were inoculated by infiltrating with the bacterial suspension with a concentration of OD_600_ of 0.1. The infiltrated leaf part was collected 24 h after inoculation, snap frozen in liquid nitrogen, and stored at −80°C immediately.

HR was tested in pepper leaves (*Capsicum annuum* ‘ECW‐10R’) as previously described (Castañeda et al., 2005; Li et al., 2014). Briefly, Xcc strains were cultured in NYG overnight and cells were collected, washed with 10 mM sodium phosphate buffer, and resuspended in the same buffer to a cell density of OD_600_ of 0.01. Then, the bacterial suspension was infiltrated into the abaxial side of the pepper leaves. The inoculated plants were kept in the greenhouse to observe HR symptoms and to gauge conductivity at 0, 8, 16, and 24 hpi. For conductivity measurements, samples (leaf discs of 0.4 cm^2^) were collected and soaked in 10 ml of ultrapure water with shaking at 200 rpm for 30 min. The leaf discs were then removed and the conductivity of the water was measured.

## Supporting information


**Figure S1** Western blotting of the eluted VemR::3 × FLAG fusion protein. After co‐immunoprecipitation, a western blot assay was performed to detect the eluted VemR::3 × FLAG fusion protein. Protein samples were separated by SDS‐PAGE and transferred to a PVDF membrane. The presence of the fusion proteins was detected by an anti‐FLAG‐tag mouse monoclonal antibody. 8004, the *Xanthomonas campestris* pv. *campestris* wild‐type strain; 8004/VemR::3 × FLAG, as 8004 but the VemR is fused with 3 × FLAG

**Figure S2** VemR functions downstream of HpaS in the regulatory pathway of T3SS in *Xanthomonas campestris* pv. *campestris* (Xcc). (a) Hypersensitive response (HR) induction by Xcc strains in nonhost plants. The Xcc wild‐type strain 8004, the *vemR* deletion mutant Δ*vemR*, the *hpaS* deletion mutant Δ*hapS*, the *hpaS* and *vemR* double deletion mutant Δ*hapS*Δ*vemR*, the cross‐complemented strain Δ*hpaS*/pR3F*vemR*, and the control strain Δ*hpaS*/pLAFR3 were cultured overnight. Bacterial cells were collected and resuspended in 10 mM sodium phosphate buffer to a cell density of 10^7^ cfu/ml. The bacterial resuspensions were infiltrated into pepper leaf mesophyll tissue. HR symptoms were recorded at 24 h postinoculation (hpi) (top). The electrolyte leakage in the pepper leaves inoculated with Xcc strains was tested (bottom). The conductivity of the infiltrated spots was measured at 0, 8, 16, and 24 hpi, with four 0.4 cm^2^ leaf discs collected from the infiltrated area for each sample. Three samples were taken for each measurement in each experiment. Data are shown as the mean ± *SD* of three replicates from a representative experiment. Asterisks indicate significant difference (**p < 0.01; n.s., not significant) when the mutant strain compared with the wild‐type strain 8004 by analysis of variance (ANOVA) and Dunnett’s post hoc test. Similar results were obtained in two other independent experiments. (b) The expression levels of *hrp* genes in the Xcc wild‐type strain 8004, the *hpaS* deletion mutant Δ*hapS*, the *hpaS* and *vemR* double deletion mutant Δ*hapS*Δ*vemR*, and the cross‐complemented strain Δ*hpaS*/pR3F*vemR* in the host plant. The strains were inoculated into the leaves of Chinese radish by infiltration with a syringe. The infiltrated leaf part was collected 24 hpi, and total RNA was extracted and reverse transcription‐quantitative PCR was performed. Values given are the mean ± *SD* of triplicate measurements from a representative experiment. Differences were evaluated using ANOVA and Dunnett’s post hoc test (**p < 0.01; n.s., not significant). Similar results were obtained in two other independent experiments

**Table S1** Bacterial strains and plasmids used in this work

**Table S2** The ≥2‐fold differentially expressed genes of the *vemR*‐mutant strain cultured in the NYG medium

**Table S3** Primers used in this work

**Table S4** The potential VemR‐interacting proteins identified by co‐immunoprecipitation coupled with LC–MS/MS assays

## Data Availability

The data that support the findings of this study are available from the corresponding author upon reasonable request.
