# Supplementary figures and images for: Xanthomonas campestris VemR enhances the transcription of the T3SS key regulator HrpX via physical interaction with HrpG
Source: Mol Plant Pathol. 2023 Jan 10;24(3):232–47. doi: 10.1111/mpp.13293 (PMC9923393; doi:10.1111/mpp.13293)

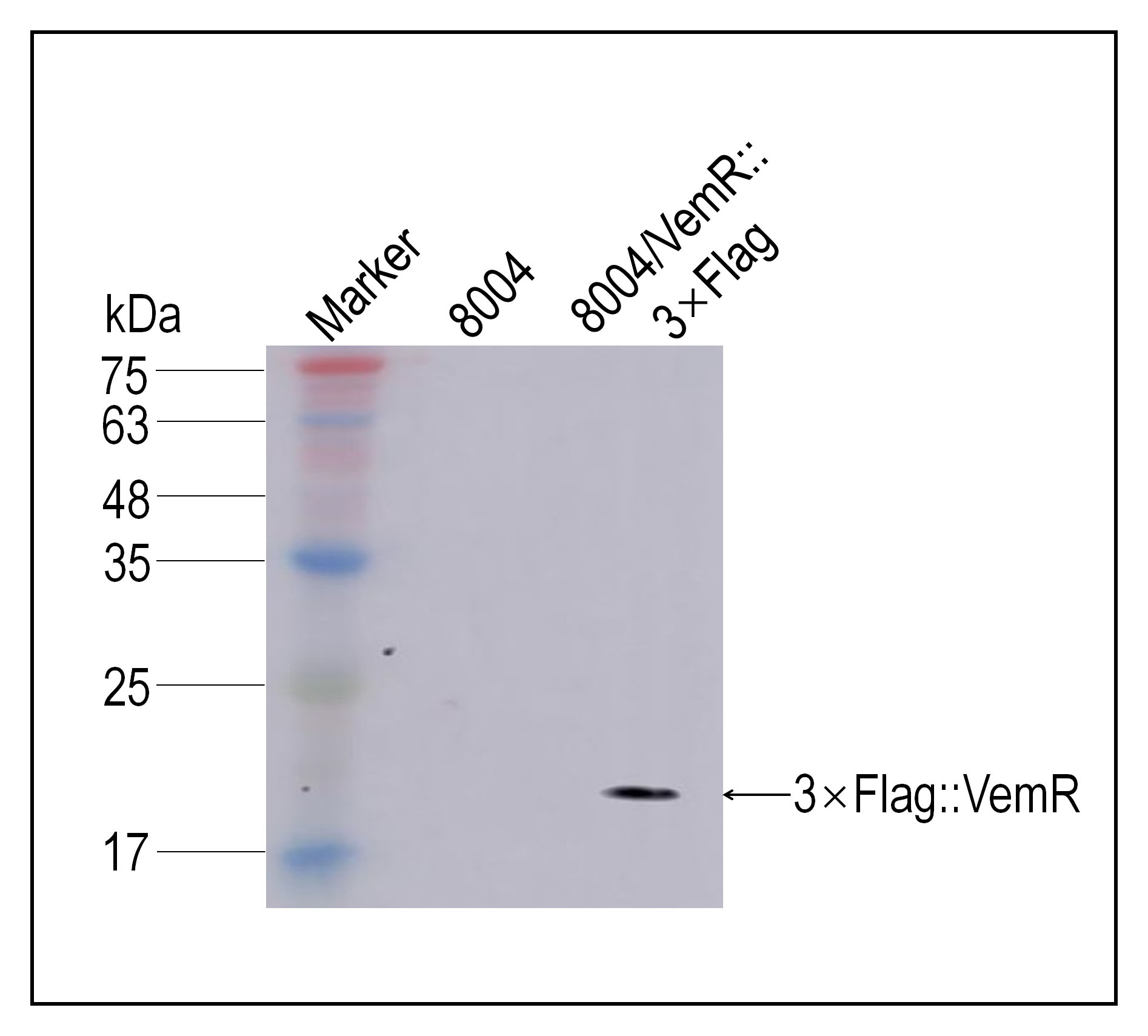

Supplement: Supplementary file 1 — Figure S1 Western blotting of the eluted VemR::3 × FLAG fusion protein. After co‐immunoprecipitation, a western blot assay was performed to detect the eluted VemR::3 × FLAG fusion protein. Protein samples were separated by SDS‐PAGE and transferred to a PVDF membrane. The presence of the fusion proteins was detected by an anti‐FLAG‐tag mouse monoclonal antibody. 8004, the Xanthomonas campestris pv. campestris wild‐type strain; 8004/VemR::3 × FLAG, as 8004 but the VemR is fused with 3 × FLAG [file MPP-24-232-s004.jpg]

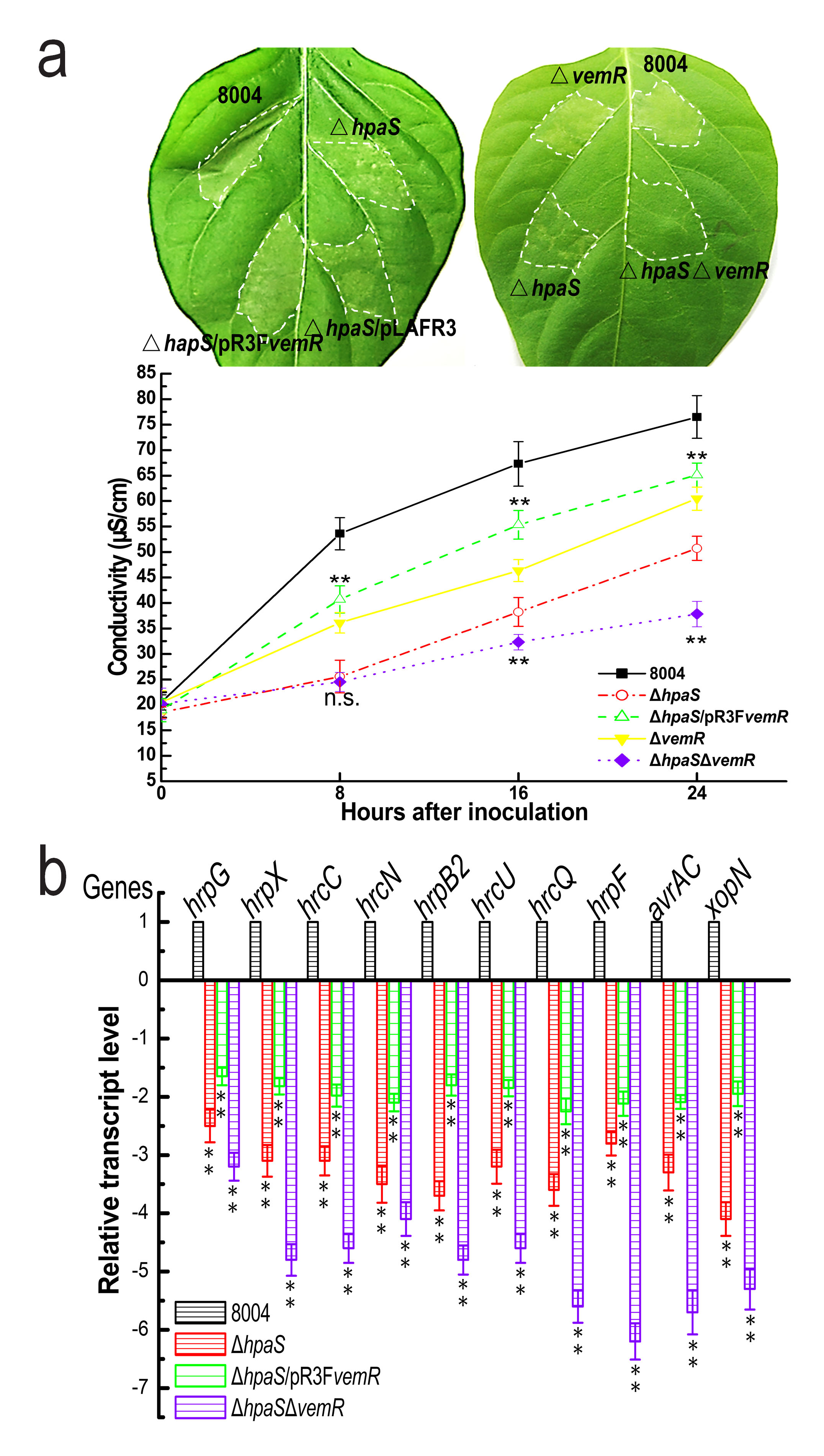

Supplement: Supplementary file 2 — Figure S2 VemR functions downstream of HpaS in the regulatory pathway of T3SS in Xanthomonas campestris pv. campestris (Xcc). (a) Hypersensitive response (HR) induction by Xcc strains in nonhost plants. The Xcc wild‐type strain 8004, the vemR deletion mutant ΔvemR, the hpaS deletion mutant ΔhapS, the hpaS and vemR double deletion mutant ΔhapSΔvemR, the cross‐complemented strain ΔhpaS/pR3FvemR, and the control strain ΔhpaS/pLAFR3 were cultured overnight. Bacterial cells were collected and resuspended in 10 mM sodium phosphate buffer to a cell density of 107 cfu/ml. The bacterial resuspensions were infiltrated into pepper leaf mesophyll tissue. HR symptoms were recorded at 24 h postinoculation (hpi) (top). The electrolyte leakage in the pepper leaves inoculated with Xcc strains was tested (bottom). The conductivity of the infiltrated spots was measured at 0, 8, 16, and 24 hpi, with four 0.4 cm2 leaf discs collected from the infiltrated area for each sample. Three samples were taken for each measurement in each experiment. Data are shown as the mean ± SD of three replicates from a representative experiment. Asterisks indicate significant difference (**p < 0.01; n.s., not significant) when the mutant strain compared with the wild‐type strain 8004 by analysis of variance (ANOVA) and Dunnett’s post hoc test. Similar results were obtained in two other independent experiments. (b) The expression levels of hrp genes in the Xcc wild‐type strain 8004, the hpaS deletion mutant ΔhapS, the hpaS and vemR double deletion mutant ΔhapSΔvemR, and the cross‐complemented strain ΔhpaS/pR3FvemR in the host plant. The strains were inoculated into the leaves of Chinese radish by infiltration with a syringe. The infiltrated leaf part was collected 24 hpi, and total RNA was extracted and reverse transcription‐quantitative PCR was performed. Values given are the mean ± SD of triplicate measurements from a representative experiment. Differences were evaluated using ANOVA and Dunnett’s post hoc [file MPP-24-232-s005.jpg]
