# Supplementary material for: Xanthomonas campestris VemR enhances the transcription of the T3SS key regulator HrpX via physical interaction with HrpG
Source: Mol Plant Pathol. 2023 Jan 10;24(3):232–47. doi: 10.1111/mpp.13293 (PMC9923393; doi:10.1111/mpp.13293)
Supplement: Supplementary file 3 — Table S1 Bacterial strains and plasmids used in this work [file MPP-24-232-s006.doc]

**Table S1. Bacterial strains and plasmids used in this study**

| **Strains or plasmids** | **Relevant characteristics** | **Reference or source** |
| --- | --- | --- |
| ***E. coli* strains** |  |  |
| JM109 | *RecA*1*, endA*1*, gyrA*96*, thi, supE*44*, relA*1  △ (*lac-proAB*)/F’ [*traD36, lacI*^q^, *lacZ* △M15] | Yanisch-Perron et al., 1985 |
| DH5α | Φ80△*lacZM*15 *recA1 endA1 deoR* | Gibco BRL, Life Technologies |
| M15 | *lac ara gal mtl recA1 uvr1* [pREP4 *lacI* Kan^r^] | Qiagen |
| BL21(DE3) | F^-^ *ompT gal dcm lon hsdS_B_* (*r^-^_B_ m^-^_B_*) λ(DE3) | Novagen, Germany |
| M15/pQE-30-VemR | M15 harboring recombinant plasmid pQE-30-VemR | Li et al., 2020 |
| M15/pQE-30-VemR_D11/56A_ | M15 harboring recombinant plasmid pQE-30-VemR_D11/56A_. | Li et al., 2020 |
| BL21/pET-30a-McvR | BL21(DE3) harboring recombinant plasmid pET-30a-McvR | Li et al., 2022 |
| BL21/pET-32a-HupB | BL21(DE3) harboring recombinant plasmid pET-32a-HupB | Li et al., 2022 |
| M15/pQE-HrpG | M15 harboring recombinant plasmid pQE-HrpG | Li et al., 2014 |
| BL21/pGEX-HrpG | BL21(DE3) harboring recombinant plasmid pGEX-HrpG | This work |
| XL1-Blue MRF' | Reporter strain, Δ*(mcrA)183* Δ*(mcrCB-hsdSMR-mrr)173 endA1 hisB supE44 thi-1 recA1 gyrA96relA1 lac* [F*´ lacIq HIS3 aadA* Kan*^r^*] | Stratagene |
| XL1-Blue MRF'/pBT*vemR* /pTRG | XL1-Blue MRF' harboring plasmids pBT*vemR* and pTRG | This work |
| XL1-Blue MRF'/pBT*vemR* /pTRG*hrpG* | XL1-Blue MRF' harboring plasmids pBT*vemR* and pTRG*hrpG* | This work |
| XL1-Blue MRF'/pBT /pTRG*hrpG* | XL1-Blue MRF' harboring plasmids pBT and pTRG*hrpG* | This work |
| XL1-Blue MRF'/ pBTPrtA/pTRGMip | XL1-Blue MRF' harboring plasmids pBTPrtA and pTRGMip | Meng et al.,2011 |
| ***Xanthomonas campestris* pv. *campestris* strains** |  |  |
| 8004 | Wild-type strain. Rif^r^ | Daniels et al., 1984 |
| 8004/VemR::3×Flag | As 8004, but chromosomally encoding a 3×Flag fused VemR protein. Rif^r^ | This work |
| Δ*vemR* | As 8004, but *vemR* gene (*XC_2252*) deleted. Rif^r^ | Li et al., 2020 |
| CΔ*vemR* | Δ*vemR* harboring a recombinant plasmid pR3O*vemR* derived from the ORF of *vemR* cloned into the plasmid pLAFR3. Rif^r^ Tet^r^ | Li et al., 2020 |
| Δ*hrpG* | As 8004, but *hrpG* gene (*XC_3077*) deleted. Rif^r^ | This work |
| ∆*hrpG*AvrAC::3×Flag | Δ*hrpG* chromosomally encoding a 3×Flag fused AvrAC protein. Rif^r^ | This work |
| ∆*hrpG*XopN::3×Flag | Δ*hrpG* chromosomally encoding a 3×Flag fused XopN protein. Rif^r^ | This work |
| ∆*hrpG*∆*vemR* | As 8004, but *hrpG* and *vemR* deleted. Rif^r^. Rif^r^ | This work |
| ∆*hrpG*∆*vemR*AvrAC::3×Flag | Δ*hrpG*∆*vemR* chromosomally encoding a 3×Flag fused AvrAC protein. Rif^r^ | This work |
| ∆*hrpG*∆*vemR*XopN::3×Flag | Δ*hrpG*∆*vemR* chromosomally encoding a 3×Flag fused XopN protein. Rif^r^ | This work |
| ∆*hrpG*AvrAC::Flag/pR3G | ∆*hrpG*AvrAC::Flag harboring a recombinant plasmid pR3G derived from the promoterless *hrpG* cloned into the plasmid pLAFR3. Rif^r^ Tet^r^ | This work |
| ∆*hrpG*∆*vemR*AvrAC::Flag/pR3G | ∆*hrpG*∆*vemR*AvrAC::Flag harboring a recombinant plasmid pR3G. Rif^r^ Tet^r^ | This work |
| ∆*hrpG*XopN::Flag/pR3G | ∆*hrpG*XopN::Flag harboring a recombinant plasmid pR3G. Rif^r^ Tet^r^ | This work |
| ∆*hrpG*∆*vemR*XopN::Flag/pR3G | ∆*hrpG*∆*vemR*XopN::Flag harboring a recombinant plasmid pR3G. Rif^r^ Tet^r^ | This work |
| Δ*vemR*/pR3G | ΔvemR harboring a recombinant plasmid pR3G. Rif^r^ Tet^r^ | This work |
| Δ*vemR*/pLAFR3 | ΔvemR harboring an empty vector pLAFR3. Rif^r^ Tet^r^ | This work |
| Δ*hrcV* | As 8004, but *hrcV* deleted. Rif^r^ | Author’s lab collection |
| Δ*hpaS* | As 8004, but *hpaS* gene (*XC_3670*) deleted. Rif^r^ Kan^r^ | Li et al., 2014 |
| Δ*hpaS*/pR3F*vemR* | ∆*hpaS* harboring a recombinant plasmid pR3F*vemR*, Rif^r^ Kan^r^ Tet^r^ | Li et al., 2020 |
| ∆*hpaS*/pLAFR3 | ΔhpaS harboring an empty vector pLAFR3. Rif^r^ Kan^r^ Tet^r^ | Li et al., 2020 |
| Δ*hpaS*Δ*vemR* | As 8004, but *hpaS* and *vemR* genes deleted. Rif^r^ Gm^r^ | Li et al., 2020 |
| **Plasmids** |  |  |
| pLAFR3 | Broad host range cloning vector, Tet^r^ | Staskawicz et al., 1987 |
| pRK2073 | Helper plasmid, Tra+, Mob+, ColE1, Spc^r^. | Leong et al., 1982 |
| pK18*mobsacB* | pUC18 derivative, *lacZα*, *sacB*, Kan^r^, *mob* site. Allelic exchange vector (Suicidal vector carrying *sacB* gene for mutagenesis) | Schäfer et al., 1994 |
| pK18*mobsacBhrpG* | pK18*mobsacB* containing fragments flanking *hrpG*. Kan^r^ | This work |
| pK*vemR*::*flag* | pK18*mobsacB* containing fragment composing 381-bp VemR-coding sequence, 66-bp 3×Flag-coding sequence, 3-bp stop codon and 373-bp downstream of the *vemR*. | This work |
| pK*avrAC*::*flag* | pK18*mobsacB* containing fragment composing 387-bp AvrAC-coding sequence, 66-bp 3×Flag-coding sequence, 3-bp stop codon and 414-bp downstream of the *avrAC*. | This work |
| pK*xopN*::*flag* | pK18*mobsacB* containing fragment composing 391-bp XopN-coding sequence, 66-bp 3×Flag-coding sequence, 3-bp stop codon and 393-bp downstream of the *xopN*. | This work |
| pR3O*vemR* | 381-bp DNA fragment of the *vemR* gene coding sequence (*XC_2252*) of *Xcc* strain cloned into the plasmid pLAFR3. Tet^r^ | Li et al., 2020 |
| pQE-30 | Expression vector, allowing the production of fusion proteins containing amino terminal 6×His-tagged sequences. Ampr | Qiagen |
| pQE-30-VemR | pQE-30 containing a 381-bp fragment of *vemR* gene coding region | This work |
| pQE-30-VemR_D11/56A_ | pQE-30 containing a 381-bp fragment of point-mutated *vemR* gene ( replacing aspartate at position 11 and 56 to alanine) | This work |
| pET-30a | Expression vector, allow the production of fusion proteins containing amino terminal 6×His-tagged sequences. Kanr | Novagen |
| pET-30a -McvR | pET-30a containing a 369-bp fragment of *mcvR* gene coding region | Li et al., 2022 |
| pET-32a | Expression vector, allow the production of fusion proteins containing amino terminal thioredoxin-tagged and carboxyl-terminal 6×His-tagged sequences. Amp^r^ | Novagen |
| pET-32a-HupB | pET-32a containing a *hupB* gene coding sequence | Authors lab collection |
| pQE-30 Xa | Expression vector, allow the production of fusion proteins containing amino terminal 6xHis-tagged sequences. Ampr | Qiagen |
| pQE-HrpG | pQE-30 Xa containing a 789-bp fragment of *hrpG* coding region. | Li et al., 2014 |
| pGEX-4T-1 | Expression vector, allow the production of fusion proteins containing amino terminal GST-tagged sequences. Amp^r^ | GE Healthcare |
| pGEX-HrpG | pGEX-4T-1 containing a 789-bp fragment of *hrpG* coding region. | This work |
| pR3G | pLAFR3 containing an 899-bp fragment including promoterless *hrpG* gene, Tet^r^ | Authors lab collection |
| pBT | Two-hybrid system bait plasmid containing the *cat* gene, p15A origin of replication and λ cI ORF. | Stratagene |
| pBT*vemR* | pBT derivative carrying 381-bp fragment of *vemR* gene coding region. Cat^r^ | This work |
| pTRG | Two-hybrid system target plasmid containing the *tet* gene, ColE1 origin of replication, and RNA polymerase α subunit ORF. | Stratagene |
| pTRG*hrpG* | pTRG derivative carrying the full length of the coding region of *hrpG* gene (789-bp). Tet^r^ | This work |
| pR3F*vemR* | 434-bp DNA fragment of the *vemR* gene (including 50 nucleotides upstream of the start codon) of *Xcc* strain cloned into the plasmid pLAFR3. Tet^r^ | Li et al., 2020 |

**References**

Daniels, M.J., Barber, C.E., Turner, P.C., Sawczyc, M.K., Byrde, R.J.W., and Fielding, A.H. (1984) Cloning of genes involved in pathogenicity of *Xanthomonas campestris* pv. *campestris* using the broad host range cosmid pLAFR1. *EMBO J* **3:** 3323–3328.

Leong, S.A., Ditta, G.S., and Helinski, D.R. (1982) Heme biosynthesis in *Rhizobium*. Identification of a cloned gene coding for delta-aminolevulinic acid synthetase from *Rhizobium meliloti*. *J Biol Chem* **257:** 8724–8730.

Li, R.F., Lu, G.T., Li, L., Su, H.Z., Feng, G.F., Chen, Y., *et al*. (2014) Identification of a putative cognate sensor kinase for the two-component response regulator HrpG, a key regulator controlling the expression of the *hrp* genes in *Xanthomonas campestris* pv. *campestris*. *Environ Microbiol* **16**: 2053–2071.

Li, R.F., Ren, P.D., Liu, Q.Q., Yao, J.L., Wu, L., Zhu, G.N., et al. (2022). McvR, a single domain response regulator regulates motility and virulence in the plant pathogen Xanthomonas campestris. *Molecular plant pathology* **23:** 649–663.

Li, R.F., Wang, X.X., Wu, L., Huang, L., Qin, Q.J., Yao, J.L., et al. (2020). Xanthomonas campestris sensor kinase HpaS co-opts the orphan response regulator VemR to form a branched two-component system that regulates motility. *Molecular plant pathology* **21:** 360–375.

Meng, Q. L., Tang, D. J., Fan, Y. Y., Li, Z. J., Zhang, H., He, Y. Q., Jiang, B. L., Lu, G. T., & Tang, J. L. (2011). Effect of interactions between Mip and PrtA on the full extracellular protease activity of Xanthomonas campestris pathovar campestris. *FEMS microbiology letters*, **323**: 180–187.

Schäfer, A., Tauch, A., Jäger, W., Kalinowski, J., Thierbach, G., and Pühler, A. (1994) Small mobilizable multi-purpose cloning vectors derived from the *Escherichia coli* plasmids pK18 and pK19: selection of defined deletions in the chromosome of *Corynebacterium glutamicum*. *Gene* **145:** 69–73.

Staskawicz, B., Dahlbeck, D., Keen, N., and Napoli, C. (1987) Molecular characterization of cloned avirulence genes fromrace 0 and race 1 of *Pseudomonas syringae* pv. *glycinea*. *J Bacteriol* **169:** 5789–5794.

Yanisch-Perron, C., Vieira, J., and Messing, J. (1985) Improved M13 phage cloning vectors and host strains: nucleotide sequences of the M13mp18 and pUC19 vectors. *Gene* **33**: 103–119.
