# Supplementary material for: Xanthomonas campestris VemR enhances the transcription of the T3SS key regulator HrpX via physical interaction with HrpG
Source: Mol Plant Pathol. 2023 Jan 10;24(3):232–47. doi: 10.1111/mpp.13293 (PMC9923393; doi:10.1111/mpp.13293)
Supplement: Supplementary file 4 — Table S2 The ≥2‐fold differentially expressed genes of the vemR‐mutant strain cultured in the NYG medium [file MPP-24-232-s003.doc]

**Table S2** The ≥2-fold differentially expressed genes of the *vemR*-mutant strain cultured in the NYG medium

| **Function Category** | **Gene ID** | **Name** | **Annotation** | **fold change**  ***vemR*^-^/wt** |
| --- | --- | --- | --- | --- |
| **Amino acids biosynthesis**  **(1)** | *XC_0548* | *aroG* | phospho-2-dehydro-3-deoxyheptonate aldolase, phe-sensitive | -2.44 |
| **Biosynthesis of cofactors, prosthetic groups, carriers**  **(6)** | *XC_0400* | *bioB* | biotin synthase | -8.03 |
|  | *XC_3952* | *entF* | ATP-dependent serine activating enzyme | -2.73 |
|  | *XC_0983* | *cysG* | siroheme synthase | 2.13 |
|  | *XC_1169* | *pqqE* | PqqE protein | 5.22 |
|  | *XC_3644* | *ptps* | 6-pyruvoyl tetrahydrobiopterin synthase | 2.21 |
|  | *XC_3751* | *entB* | isochorismatase-like protein | 20.38 |
| **Cell envelope and cell structure**  **(36)** | *XC_0504* |  | transmembrane protein | -2.47 |
|  | *XC_0937* | *pilQ* | fimbrial assembly protein | -4.69 |
|  | *XC_0938* | *pilP* | fimbrial assembly protein | -5.42 |
|  | *XC_0939* | *pilO* | fimbrial assembly membrane protein | -4.21 |
|  | *XC_0940* | *pilN* | fimbrial assembly membrane protein | -5.04 |
|  | *XC_0941* | *pilM* | fimbrial assembly membrane protein | -5.00 |
|  | *XC_1059* |  | pilin | -2.97 |
|  | *XC_1183* | *pilG* | pilus protein | -2.97 |
|  | *XC_1184* | *pilH* | PilH protein | -5.89 |
|  | *XC_1185* | *pilI* | pilus biogenesis protein | -3.82 |
|  | *XC_1186* | *pilJ* | pilus biogenesis protein | -4.24 |
|  | *XC_1187* | *pilL* | PilL protein | -4.03 |
|  | *XC_1358* | *pilT* | twitching motility protein | -4.10 |
|  | *XC_1359* | *pilU* | twitching motility protein | -5.71 |
|  | *XC_1621* | *fimT* | pre-pilin like leader sequence | -7.73 |
|  | *XC_1622* | *pilV* | pre-pilin leader sequence | -5.78 |
|  | *XC_1624* | *pilX* | PilX protein | -8.13 |
|  | *XC_1626* | *pilE1* | type IV pilin | -5.96 |
|  | *XC_3640* | *uptC* | type II secretion system protein-like protein | -4.86 |
|  | *XC_3823* | *pilA* | fimbrial protein | -3.48 |
|  | *XC_4168* | *ndvB* | NdvB protein | -2.61 |
|  | *XC_4327* | *oar* | Oar protein | -6.69 |
|  | *XC_0706* | *mltB* | membrane-bound lytic transglycosylase | 2.21 |
|  | *XC_1176* | *ponB* | penicillin-binding protein 1B | 2.16 |
|  | *XC_1816* | *amiC* | N-acetylmuramoyl-L-alanine amidase | 3.23 |
|  | *XC_2796* | *oprN* | outer membrane protein | 3.73 |
|  | *XC_2802* |  | outer membrane protein | 2.38 |
|  | *XC_2815* | *pcp* | peptidoglycan-associated outer membrane lipoprotein | 2.59 |
|  | *XC_3129* | *yjdB* | inner membrane protein | 16.52 |
|  | *XC_3510* | *murG* | UDP-N-acetylglucosamine-N- acetylmuramyl-(pentapeptide) pyrophosphoryl-undecaprenol N-acetylglucosamine transferase | 2.32 |
|  | *XC_3512* | *mraY* | phospho-N-acetylmuramoyl-pentapeptide- transferase | 2.22 |
|  | *XC_3513* | *murF* | UDP-N-acetylmuramoylalanyl-D-glutamyl-2, 6-diaminopimelate-D-alanyl-D-alanyl ligase | 2.84 |
|  | *XC_3514* | *murE* | UDP-N-acetylmuramoylalanyl-D- glutamate-2,6-diaminopimelate ligase | 2.45 |
|  | *XC_3638* | *uptE* | outer membrane protein | 2.10 |
|  | *XC_3694* | *ompW* | outer membrane protein | 2.67 |
|  | *XC_4085* | *ank2* | ankyrin-like protein | 3.12 |
| **Cellular processes**  **(52)** | *XC_0638* | *tsr* | chemotaxis protein | -2.02 |
|  | *XC_1409* | *cheB* | protein-glutamate methylesterase | -4.75 |
|  | *XC_1410* | *cheR* | response regulator for chemotaxis | -5.23 |
|  | *XC_1412* | *cheW* | chemotaxis protein | -4.76 |
|  | *XC_1413* | *mcp* | chemotaxis protein | -12.69 |
|  | *XC_1414* | *cheA* | chemotaxis histidine protein kinase | -4.55 |
|  | *XC_1801* | *mcp* | chemotaxis protein | -3.83 |
|  | *XC_1937* | *tlpC* | methyl-accepting chemotaxis protein | -6.86 |
|  | *XC_2223* | *mcp* | chemotaxis protein | -2.21 |
|  | *XC_2245* | *fliC* | flagellar protein | -3.79 |
|  | *XC_2302* | *cheY* | chemotaxis response regulator | -2.54 |
|  | *XC_2303* | *cheA* | chemotaxis protein | -2.00 |
|  | *XC_2306* | *tsr* | chemotaxis protein | -6.37 |
|  | *XC_2309* | *tsr* | chemotaxis protein | -7.17 |
|  | *XC_2311* | *tsr* | chemotaxis protein | -2.80 |
|  | *XC_2314* | *tsr* | chemotaxis protein | -2.40 |
|  | *XC_2315* |  | methyl-accepting chemotaxis protein | -3.11 |
|  | *XC_2316* | *tsr* | chemotaxis protein | -2.24 |
|  | *XC_2318* | *cheW* | chemotaxis protein | -5.40 |
|  | *XC_2320* | *tsr* | chemotaxis protein | -17.97 |
|  | *XC_2321* | *cheR* | chemotaxis protein methyltransferase | -5.17 |
|  | *XC_2234* | *flgB* | flagellar protein | 7.66 |
|  | *XC_2235* | *flgC* | flagellar biosynthesis, cell-proximal portion of basal-body rod | 4.75 |
|  | *XC_2236* | *flgD* | flagellar protein | 5.35 |
|  | *XC_2237* | *flgE* | flagellar biosynthesis, hook protein | 4.71 |
|  | *XC_2238* | *flgF* | flagellar protein | 4.00 |
|  | *XC_2239* | *flgG* | flagellar biosynthesis, cell-distal portion of basal-body rod | 2.42 |
|  | *XC_2240* | *flgH* | flagellar L-ring protein | 3.01 |
|  | *XC_2241* | *flgI* | flagellar protein | 3.71 |
|  | *XC_2242* | *flgJ* | flagellar protein | 3.87 |
|  | *XC_2243* | *flgK* | flagellar protein | 3.16 |
|  | *XC_2244* | *flgL* | flagellar protein | 2.86 |
|  | *XC_2259* | *fliE* | flagellar protein | 7.90 |
|  | *XC_2260* | *fliF* | flagellar protein | 3.56 |
|  | *XC_2261* | *fliG* | flagellar protein | 2.80 |
|  | *XC_2262* | *fliH* | flagellar protein | 4.12 |
|  | *XC_2263* | *fliI* | flagellar protein | 4.58 |
|  | *XC_2264* | *fliJ* | flagellar FliJ protein | 2.91 |
|  | *XC_2265* | *fliK* | flagellar protein | 3.03 |
|  | *XC_2266* | *fliL* | flagellar biosynthesis protein | 26.50 |
|  | *XC_2267* | *fliM* | flagellar protein | 20.10 |
|  | *XC_2268* | *fliN* | flagellar protein | 18.64 |
|  | *XC_2269* | *fliO* | flagellar protein | 25.35 |
|  | *XC_2270* | *fliP* | flagellar biosynthetic protein | 23.48 |
|  | *XC_2272* | *fliQ* | flagellar biosynthesis | 2.72 |
|  | *XC_2273* | *fliR* | flagellar biosynthetic protein | 2.50 |
|  | *XC_2277* | *flhB* | flagellar protein | 2.21 |
|  | *XC_2278* | *flhA* | flagellar biosynthetic protein FlhA | 2.29 |
|  | *XC_2279* | *flhF* | flagellar biosynthetic protein | 4.38 |
|  | *XC_2282* | *cheY* | chemotaxis protein | 2.15 |
|  | *XC_2283* | *cheZ* | chemotaxis related protein | 2.41 |
|  | *XC_2284* | *cheA* | chemotaxis related protein | 2.72 |
| **Central intermediary metabolism**  **(11)** | *XC_0158* |  | xylosidase/arabinosidase | -2.37 |
|  | *XC_1221* | *fucA1* | alpha-L-fucosidase | -6.73 |
|  | *XC_1642* | *aglA* | alpha-glucosidase | -3.65 |
|  | *XC_2477* | *xylA* | xylose isomerase | -5.39 |
|  | *XC_2478* |  | D-xylulokinase | -4.52 |
|  | *XC_2480* | *xylS* | alpha-xylosidase | -22.18 |
|  | *XC_2482* |  | sialic acid-specific 9-O-acetylesterase | -5.61 |
|  | *XC_2487* | *dgoA* | 4-hydroxy-2-oxoglutarate aldolase/2-deydro-3-deoxyphosphogluconate aldolase | -7.20 |
|  | *XC_2985* | *bga* | beta-galactosidase | -3.26 |
|  | *XC_2325* | *pcaD or catD* | beta-ketoadipate enol-lactone hydrolase | 5.74 |
|  | *XC_3767* |  | UDP-glucose 4-epimerase | 18.38 |
| **Energy and carbon metabolism**  **(15)** | *XC_1300* |  | quinol oxidase, subunit I | 3.96 |
|  | *XC_1301* | *qxtB* | quinol oxidase, subunit II | 5.70 |
|  | *XC_1384* |  | alcohol dehydrogenase | 11.30 |
|  | *XC_1445* | *mocA* | oxidoreductase | 3.33 |
|  | *XC_1446* | *mocA* | oxidoreductase | 5.15 |
|  | *XC_1884* | *cydB* | cytochrome D ubiquinol oxidase subunit II | 2.10 |
|  | *XC_2188* | *fdh* | glutathione-dependent formaldehyde dehydrogenase | 8.52 |
|  | *XC_2659* | *gcd* | glucose dehydrogenase | 16.64 |
|  | *XC_2800* | *dauE or aknU* | aklaviketone reductase | 3.75 |
|  | *XC_3167* |  | oxidoreductase | 10.91 |
|  | *XC_3170* |  | oxidoreductase | 19.73 |
|  | *XC_3740* |  | oxidoreductase | 9.72 |
|  | *XC_3766* |  | oxidoreductase | 29.09 |
|  | *XC_3774* | *Zn-dependent* | Zn-dependent alcohol dehydrogenase | 19.12 |
|  | *XC_3896* | *coxD* | cytochrome C oxidase assembly factor | 2.58 |
| **Fatty acid and phospholipidmeatbolism**  **(1)** | *XC_0707* | *rlpA* | rare lipoprotein A | 2.24 |
| **Regulatory functions**  **(6)** | *XC_0092* | *moxR* | methanol dehydrogenase regulator | -3.90 |
|  | *XC_0295* |  | transcriptional regulator lysR family | -2.03 |
|  | *XC_0778* |  | transcriptional regulator | -2.44 |
|  | *XC_3197* | *pilH* | regulatory protein-PilH family | -2.61 |
|  | *XC_3758* | *exsF* | regulatory protein | 3.81 |
|  | *XC_4254* | *slyA* | transcriptional regulator for cryptic hemolysin | 2.02 |
| **Replication and DNA metabolism**  **(3)** | *XC_0132* |  | deoxycytidylate deaminase | -8.47 |
|  | *XC_0355* |  | site-specific recombinase | -3.93 |
|  | *XC_4288* | *recB* | exodeoxyribonuclease V beta chain | 2.06 |
| **Transport**  **(33)** | *XC_0124* | *iroN* | TonB-dependent receptor | -3.89 |
|  | *XC_0756* | *fyuA* | TonB-dependent receptor | -3.97 |
|  | *XC_0759* | *btuB* | TonB-dependent receptor | -5.23 |
|  | *XC_1165* | *fepA* | TonB-dependent receptor | -4.22 |
|  | *XC_1222* | *iroN* | TonB-dependent receptor | -10.46 |
|  | *XC_1241* | *btuB* | TonB-dependent receptor | -3.91 |
|  | *XC_1332* | *comEA* | DNA transport competence protein | -6.03 |
|  | *XC_1546* | *btuB* | TonB-dependent receptor | -2.11 |
|  | *XC_1644* | *btuB* | TonB-dependent receptor | -3.03 |
|  | *XC_1772* | *potF* | periplasmic putrescine-binding protein; permease protein | -2.15 |
|  | *XC_2296* | *cirA* | TonB-dependent receptor | -3.42 |
|  | *XC_2476* | *xylE* | MFS transporter | -4.52 |
|  | *XC_2484* |  | TonB-dependent receptor | -60.89 |
|  | *XC_2485* | *fhuA* | TonB-dependent receptor | -4.38 |
|  | *XC_2983* | *btuB* | TonB-dependent receptor | -5.19 |
|  | *XC_3209* | *fyuA* | TonB-dependent receptor | -2.23 |
|  | *XC_0167* | *fpvA* | ferripyoverdine receptor | 2.38 |
|  | *XC_0674* | *rmrB* | MFS transporter | 5.86 |
|  | *XC_0762* | *betT* | high-affinity choline transport | 5.23 |
|  | *XC_0988* | *fecA* | TonB-dependent receptor | 3.64 |
|  | *XC_1444* | *araJ* | MFS transporter | 6.12 |
|  | *XC_1886* | *strW* | transport protein | 3.19 |
|  | *XC_1887* | *cydC* | ABC transporter ATP-binding protein | 3.47 |
|  | *XC_1947* | *smf2* | manganese transport protein | 8.07 |
|  | *XC_1951* | *yggB* | small conductance mechanosensitive ion channel | 2.51 |
|  | *XC_1989* | *ycfV* | ABC transporter ATP-binding protein | 2.09 |
|  | *XC_2143* |  | transport protein | 2.95 |
|  | *XC_2546* |  | MFS transporter | 5.50 |
|  | *XC_2354* | *feoB* | ferrous iron transport protein B | 2.17 |
|  | *XC_2928* | *catA* | cation transport protein | 6.94 |
|  | *XC_3463* | *phuR* | outer membrane hemin receptor | 3.57 |
|  | *XC_3532* | *kdpA* | potassium-transporting ATPase A chain | 11.02 |
|  | *XC_4044* | *piuB* | iron-uptake factor | 2.12 |
| **Translation**  **(21)** | *XC_0094* | *tldD* | TldD protein | -4.52 |
|  | *XC_0096* | *tldD* | TldD protein | -3.73 |
|  | *XC_0240* | *dcp* | peptidyl-dipeptidase | -3.18 |
|  | *XC_0253* |  | dipeptidyl anminopeptidase | -3.42 |
|  | *XC_0997* | *pepN* | aminopeptidase N | -3.10 |
|  | *XC_1291* |  | endoproteinase Arg-C | -3.43 |
|  | *XC_1442* |  | extracellular serine protease | -3.13 |
|  | *XC_3327* | *rpsN* | 30S ribosomal protein S14 | -2.93 |
|  | *XC_3328* | *rplE* | 50S ribosomal protein L5 | -3.49 |
|  | *XC_3329* | *rplX* | 50S ribosomal protein L24 | -3.50 |
|  | *XC_3330* | *rplN* | 50S ribosomal protein L14 | -3.57 |
|  | *XC_3331* | *rpsQ* | 30S ribosomal protein S17 | -3.35 |
|  | *XC_3332* | *rpmC* | 50S ribosomal protein L29 | -4.95 |
|  | *XC_3333* | *rplP* | 50S ribosomal protein L16 | -3.50 |
|  | *XC_3348* | *rplL* | 50S ribosomal protein L7/L12 | -2.68 |
|  | *XC_3349* | *rplJ* | 50S ribosomal protein L10 | -2.80 |
|  | *XC_3550* |  | serine protease | -6.86 |
|  | *XC_3575* |  | protease | -10.29 |
|  | *XC_0077* |  | metalloprotease | 9.58 |
|  | *XC_1572* | *truA* | tRNA pseudouridine synthase A | 4.97 |
|  | *XC_2148* |  | metallopeptidase | 7.45 |
| **Transcription**  **(5)** | *XC_2251* | *rpoN* | RNA polymerase sigma-54 factor | -2.61 |
|  | *XC_3346* | *rpoC* | RNA polymerase beta | -2.95 |
|  | *XC_0556* | *fecI* | RNA polymerase sigma factor | 2.56 |
|  | *XC_2281* | *fliA* | RNA polymerase sigma factor | 2.44 |
|  | *XC_3643* | *rhlE* | ATP-dependent RNA helicase | 2.31 |
| **Signal transduction**  **(7)** | *XC_0637* |  | histidine kinase/response regulator hybrid protein | -2.17 |
|  | *XC_1261* |  | histidine kinase/response regulator hybrid protein | -3.34 |
|  | *XC_1528* | *pilR* | two-component system regulatory protein | -2.99 |
|  | *XC_1938* |  | two-component system regulatory protein | -2.65 |
|  | *XC_1939* |  | two-component system sensor protein | -2.40 |
|  | *XC_2129* | *cvgSY* | histidine kinase/response regulator hybrid protein | -2.12 |
|  | *XC_2274* |  | GGDEF family protein | 2.28 |
| **Mobile genetic elements**  **(18)** | *XC_0144* | *IS1480* | IS1480 transposase truncated | -2.07 |
|  | *XC_0666* | *ISxac3* | ISxac3 transposase | -2.70 |
|  | *XC_0680* | *ISxac3* | ISxac3 transposase | -2.65 |
|  | *XC_0688* | *IS1480* | IS1480 transposase | -3.23 |
|  | *XC_1211* | *ISxac3* | ISxac3 transposase | -2.35 |
|  | *XC_2120* |  | major coat protein | -10.93 |
|  | *XC_2121* | *gVII* | minor coat protein | -10.61 |
|  | *XC_2124* | *gII* | phage-related protein | -4.29 |
|  | *XC_2292* | *IS1478* | IS1478 transposase | -3.01 |
|  | *XC_2392* | *IS1481* | IS1481 transposase | -2.24 |
|  | *XC_2434* |  | phage associated protein | -3.61 |
|  | *XC_2590* | *IS1481* | IS1481 transposase | -2.52 |
|  | *XC_2597* | *ISxac3* | ISxac3 transposase | -2.74 |
|  | *XC_2790* | *IS1481* | IS1481 transposase | -2.62 |
|  | *XC_3034* | *IS1495* | IS1595 transposase | -2.83 |
|  | *XC_3803* | *ISxac3* | ISxac3 transposase | -2.69 |
|  | *XC_3921* | *ISxac3* | ISxac3 transposase | -3.09 |
|  | *XC_4232* | *ISxac3* | ISxac3 transposase | -2.86 |
| **Pathogenicity and adaptation**  **(57)** | *XC_0125* |  | pectin methylesterase-like protein | -10.15 |
|  | *XC_0126* |  | pectate lyase E | -4.46 |
|  | *XC_0274* | *ohr* | organic hydroperoxide resistance protein | -2.11 |
|  | *XC_0741* | *xcsF* | type II secretion system protein F | -2.36 |
|  | *XC_0742* | *xcsG* | type II secretion system protein G | -5.38 |
|  | *XC_0783* | *celS* | cellulase S | -9.08 |
|  | *XC_1027* | *virB6* | VirB6 protein | -2.38 |
|  | *XC_1057* | *pilC* | fimbrial assembly protein | -3.12 |
|  | *XC_1119* | *bla* | beta lactamase | -5.01 |
|  | *XC_1120* | *xynB* | xylanase | -3.28 |
|  | *XC_1411* | *vieA* | response regulator | -4.78 |
|  | *XC_1625* |  | PilY1 protein | -6.28 |
|  | *XC_1632* | *virB8* | VirB8 protein | -4.11 |
|  | *XC_1633* | *virB9* | VirB9 protein | -5.33 |
|  | *XC_1634* | *virB10* | VirB10 protein | -4.80 |
|  | *XC_1635* | *virB11* | VirB11 protein | -3.67 |
|  | *XC_1636* | *virB1* | VirB1 protein | -3.22 |
|  | *XC_1637* | *virB2* | VirB2 protein | -3.03 |
|  | *XC_1638* | *virB3* | VirB3 protein | -2.79 |
|  | *XC_1639* | *virB4* | VirB4 protein | -3.83 |
|  | *XC_2016* | *virB6* | VirB6 protein | -2.25 |
|  | *XC_2637* | *MbtG* | hydroxylase | -2.99 |
|  | *XC_2820* | *gst* | glutathione S-transferase | -2.32 |
|  | *XC_2483* |  | cellulase | -36.60 |
|  | *XC_2834* | *bglS* | beta-glucosidase | -3.43 |
|  | *XC_2942* |  | serine protease | -3.90 |
|  | *XC_3024* |  | XopF1 effector | -2.03 |
|  | *XC_3576* | *xadA* | outer membrane protein | -3.66 |
|  | *XC_3379* |  | extracellular protease | -4.15 |
|  | *XC_3590* | *pel* | pectate lyase | -2.79 |
|  | *XC_3591* | *pel* | pectate lyase | -4.60 |
|  | *XC_4057* |  | TonB-like protein | -5.33 |
|  | *XC_4207* | *xynA* | endo-1,4-beta-xylanase A | -2.35 |
|  | *XC_4290* |  | hemagglutinin | -3.14 |
|  | *XC_0672* |  | multidrug resistance efflux pump | 4.04 |
|  | *XC_1754* | *yieO* | drug resistance translocase | 8.96 |
|  | *XC_1811* | *acvB* | virulence protein | 3.08 |
|  | *XC_1849* | *pglA* | polygalacturonase | 11.24 |
|  | *XC_2506* | *csrA* | carbon storage regulator | 2.04 |
|  | *XC_2798* | *mexF* | RND multidrug efflux transporter MexF | 3.09 |
|  | *XC_2799* | *mexE or acrA* | RND multidrug efflux membrane fusion protein | 3.66 |
|  | *XC_3002* | *hpa1* | Hpa1 protein | 22.49 |
|  | *XC_3003* | *hrcC* | HrcC protein | 4.72 |
|  | *XC_3004* | *hrcT* | HrpB8 protein | 4.13 |
|  | *XC_3005* | *hrpB7* | HrpB7 protein | 3.55 |
|  | *XC_3006* | *hrcN* | HrpB6 protein | 5.09 |
|  | *XC_3008* | *hrpB4* | HrpB4 protein | 4.56 |
|  | *XC_3009* | *hrcJ* | HrcJ protein | 3.81 |
|  | *XC_3011* | *hrpB1* | HrpB1 protein | 3.99 |
|  | *XC_3019* | *hrpD5* | HrpD5 protein | 4.02 |
|  | *XC_3020* | *hrpD6* | HrpD6 protein | 3.43 |
|  | *XC_3076* | *hrpX* | HrpX protein | 2.39 |
|  | *XC_3360* | *lolB* | outer membrane lipoprotein precursor | 2.16 |
|  | *XC_3754* |  | Mn-containing catalase | 67.33 |
|  | *XC_3815* |  | putative O-antigen ligase | 2.45 |
|  | *XC_3833* | *norM* | multidrug efflux protein | 2.18 |
|  | *XC_4256* | *fusE* | fusaric acid resistance protein | 3.07 |
| **Undefined category**  **(9)** | *XC_0341* | *attT* | AttT protein | -5.19 |
|  | *XC_0360* |  | transferase | -2.23 |
|  | *XC_1201* | *rebB* | RebB protein | -4.87 |
|  | *XC_2413* |  | putative NTPase VagA | -3.19 |
|  | *XC_2428* |  | ankyrin repeat protein | -3.02 |
|  | *XC_2433* |  | ATPases of the AAA+ class | -2.41 |
|  | *XC_2592* |  | RhsD protein | -3.01 |
|  | *XC_2147* |  | hydroxylase | 14.01 |
|  | *XC_3895* |  | disulphide-isomerase | 3.83 |
| **Hypothetical proteins**  **(157)** | *XC_0073* |  | conserved hypothetical protein | -3.71 |
|  | *XC_0090* |  | conserved hypothetical protein | -3.17 |
|  | *XC_0093* |  | conserved hypothetical protein | -2.52 |
|  | *XC_0129* |  | conserved hypothetical protein | -5.73 |
|  | *XC_0130* |  | conserved hypothetical protein | -6.72 |
|  | *XC_0131* |  | conserved hypothetical protein | -7.18 |
|  | *XC_0180* |  | conserved hypothetical protein | -5.69 |
|  | *XC_0251* |  | conserved hypothetical protein | -5.32 |
|  | *XC_0340* |  | conserved hypothetical protein | -3.80 |
|  | *XC_0342* |  | conserved hypothetical protein | -5.97 |
|  | *XC_0343* |  | conserved hypothetical protein | -4.38 |
|  | *XC_0344* |  | conserved hypothetical protein | -4.81 |
|  | *XC_0499* |  | conserved hypothetical protein | -3.38 |
|  | *XC_0543* |  | conserved hypothetical protein | -6.67 |
|  | *XC_0593* |  | conserved hypothetical protein | -2.15 |
|  | *XC_0603* |  | conserved hypothetical protein | -3.91 |
|  | *XC_0657* |  | conserved hypothetical protein | -2.47 |
|  | *XC_0710* |  | conserved hypothetical protein | -4.04 |
|  | *XC_0791* |  | conserved hypothetical protein | -6.73 |
|  | *XC_0792* |  | conserved hypothetical protein | -4.55 |
|  | *XC_0793* |  | conserved hypothetical protein | -5.75 |
|  | *XC_1028* |  | conserved hypothetical protein | -3.78 |
|  | *XC_1053* |  | conserved hypothetical protein | -2.65 |
|  | *XC_1188* |  | conserved hypothetical protein | -4.14 |
|  | *XC_1190* |  | conserved hypothetical protein | -5.39 |
|  | *XC_1202* |  | conserved hypothetical protein | -3.14 |
|  | *XC_1220* |  | conserved hypothetical protein | -6.05 |
|  | *XC_1231* |  | conserved hypothetical protein | -2.60 |
|  | *XC_1336* |  | conserved hypothetical protein | -3.90 |
|  | *XC_1337* |  | conserved hypothetical protein | -3.49 |
|  | *XC_1401* |  | conserved hypothetical protein | -6.00 |
|  | *XC_1415* |  | conserved hypothetical protein | -6.90 |
|  | *XC_1513* |  | conserved hypothetical protein | -2.74 |
|  | *XC_1558* |  | conserved hypothetical protein | -3.02 |
|  | *XC_1585* |  | conserved hypothetical protein | -3.12 |
|  | *XC_1623* |  | conserved hypothetical protein | -7.28 |
|  | *XC_1631* |  | conserved hypothetical protein | -4.61 |
|  | *XC_1718* |  | conserved hypothetical protein | -2.83 |
|  | *XC_1911* |  | conserved hypothetical protein | -2.33 |
|  | *XC_2015* |  | conserved hypothetical protein | -2.25 |
|  | *XC_2017* |  | conserved hypothetical protein | -3.50 |
|  | *XC_2018* |  | conserved hypothetical protein | -4.14 |
|  | *XC_2019* |  | conserved hypothetical protein | -3.01 |
|  | *XC_2122* |  | minor coat protein | -8.60 |
|  | *XC_2291* |  | conserved hypothetical protein | -2.39 |
|  | *XC_2301* |  | conserved hypothetical protein | -3.15 |
|  | *XC_2317* |  | conserved hypothetical protein | -3.73 |
|  | *XC_2319* |  | conserved hypothetical protein | -7.17 |
|  | *XC_2411* |  | conserved hypothetical protein | -3.94 |
|  | *XC_2415* |  | conserved hypothetical protein | -2.86 |
|  | *XC_2435* |  | conserved hypothetical protein | -5.86 |
|  | *XC_2479* |  | conserved hypothetical protein | -4.83 |
|  | *XC_2481* |  | conserved hypothetical protein | -16.94 |
|  | *XC_2591* |  | conserved hypothetical protein | -4.49 |
|  | *XC_2609* |  | conserved hypothetical protein | -2.19 |
|  | *XC_2740* |  | conserved hypothetical protein | -4.11 |
|  | *XC_2781* |  | conserved hypothetical protein | -3.01 |
|  | *XC_2786* |  | conserved hypothetical protein | -9.99 |
|  | *XC_2787* |  | conserved hypothetical protein | -8.82 |
|  | *XC_2788* |  | conserved hypothetical protein | -8.66 |
|  | *XC_2830* |  | conserved hypothetical protein | -2.50 |
|  | *XC_3219* |  | conserved hypothetical protein | -3.04 |
|  | *XC_3291* |  | conserved hypothetical protein | -2.01 |
|  | *XC_3540* |  | conserved hypothetical protein | -4.70 |
|  | *XC_3549* |  | conserved hypothetical protein | -9.62 |
|  | *XC_3595* |  | conserved hypothetical protein | -3.98 |
|  | *XC_3820* |  | conserved hypothetical protein | -5.36 |
|  | *XC_3955* |  | conserved hypothetical protein | -2.68 |
|  | *XC_3996* |  | conserved hypothetical protein | -2.49 |
|  | *XC_4007* |  | conserved hypothetical protein | -3.62 |
|  | *XC_4008* |  | conserved hypothetical protein | -3.69 |
|  | *XC_4129* |  | conserved hypothetical protein | -2.37 |
|  | *XC_4151* |  | conserved hypothetical protein | -2.73 |
|  | *XC_4199* |  | conserved hypothetical protein | -8.01 |
|  | *XC_4206* |  | conserved hypothetical protein | -3.05 |
|  | *XC_4217* |  | conserved hypothetical protein | -4.46 |
|  | *XC_1719* |  | hypothetical protein | -3.77 |
|  | *XC_2123* |  | hypothetical protein | -4.51 |
|  | *XC_2412* |  | hypothetical protein | -5.87 |
|  | *XC_2416* |  | hypothetical protein | -3.52 |
|  | *XC_2432* |  | hypothetical protein | -2.84 |
|  | *XC_2436* |  | hypothetical protein | -4.63 |
|  | *XC_2439* |  | hypothetical protein | -3.63 |
|  | *XC_0076* |  | conserved hypothetical protein | 10.40 |
|  | *XC_0078* |  | conserved hypothetical protein | 11.60 |
|  | *XC_0107* |  | conserved hypothetical protein | 6.44 |
|  | *XC_0217* |  | conserved hypothetical protein | 4.61 |
|  | *XC_0230* |  | conserved hypothetical protein | 6.90 |
|  | *XC_0284* |  | conserved hypothetical protein | 4.28 |
|  | *XC_0332* |  | conserved hypothetical protein | 2.71 |
|  | *XC_0414* |  | conserved hypothetical protein | 4.44 |
|  | *XC_0415* |  | conserved hypothetical protein | 2.19 |
|  | *XC_0430* |  | conserved hypothetical protein | 3.43 |
|  | *XC_0624* |  | conserved hypothetical protein | 31.49 |
|  | *XC_0632* |  | conserved hypothetical protein | 8.95 |
|  | *XC_0634* |  | conserved hypothetical protein | 5.26 |
|  | *XC_0715* |  | conserved hypothetical protein | 6.34 |
|  | *XC_0727* |  | conserved hypothetical protein | 6.23 |
|  | *XC_0921* |  | conserved hypothetical protein | 4.54 |
|  | *XC_0971* |  | conserved hypothetical protein | 10.64 |
|  | *XC_0986* |  | conserved hypothetical protein | 2.94 |
|  | *XC_0989* |  | conserved hypothetical protein | 4.04 |
|  | *XC_1177* |  | conserved hypothetical protein | 2.22 |
|  | *XC_1289* |  | conserved hypothetical protein | 43.11 |
|  | *XC_1321* |  | conserved hypothetical protein | 2.40 |
|  | *XC_1466* |  | conserved hypothetical protein | 2.54 |
|  | *XC_1493* |  | conserved hypothetical protein | 14.06 |
|  | *XC_1687* |  | conserved hypothetical protein | 48.93 |
|  | *XC_1732* |  | conserved hypothetical protein | 4.38 |
|  | *XC_1807* |  | conserved hypothetical protein | 8.68 |
|  | *XC_1883* |  | conserved hypothetical protein | 2.36 |
|  | *XC_2088* |  | conserved hypothetical protein | 2.49 |
|  | *XC_2141* |  | conserved hypothetical protein | 2.45 |
|  | *XC_2142* |  | conserved hypothetical protein | 6.10 |
|  | *XC_2144* |  | conserved hypothetical protein | 4.78 |
|  | *XC_2145* |  | conserved hypothetical protein | 5.37 |
|  | *XC_2146* |  | conserved hypothetical protein | 4.97 |
|  | *XC_2164* |  | conserved hypothetical protein | 20.82 |
|  | *XC_2189* |  | conserved hypothetical protein | 7.99 |
|  | *XC_2860* |  | conserved hypothetical protein | 2.29 |
|  | *XC_2874* |  | conserved hypothetical protein | 4.59 |
|  | *XC_2916* |  | conserved hypothetical protein | 3.04 |
|  | *XC_2921* |  | conserved hypothetical protein | 43.12 |
|  | *XC_2938* |  | conserved hypothetical protein | 7.22 |
|  | *XC_3127* |  | conserved hypothetical protein | 10.58 |
|  | *XC_3128* |  | conserved hypothetical protein | 29.62 |
|  | *XC_3171* |  | conserved hypothetical protein | 14.41 |
|  | *XC_3372* |  | conserved hypothetical protein | 2.17 |
|  | *XC_3460* |  | conserved hypothetical protein | 3.37 |
|  | *XC_3464* |  | conserved hypothetical protein | 4.51 |
|  | *XC_3711* |  | conserved hypothetical protein | 15.24 |
|  | *XC_3715* |  | conserved hypothetical protein | 10.44 |
|  | *XC_3716* |  | conserved hypothetical protein | 10.89 |
|  | *XC_3741* |  | conserved hypothetical protein | 8.03 |
|  | *XC_3752* |  | conserved hypothetical protein | 24.92 |
|  | *XC_3753* |  | conserved hypothetical protein | 66.31 |
|  | *XC_3755* |  | conserved hypothetical protein | 21.99 |
|  | *XC_3764* |  | conserved hypothetical protein | 7.84 |
|  | *XC_3772* |  | conserved hypothetical protein | 27.35 |
|  | *XC_3773* |  | conserved hypothetical protein | 32.47 |
|  | *XC_3775* |  | conserved hypothetical protein | 9.42 |
|  | *XC_3842* |  | conserved hypothetical protein | 2.40 |
|  | *XC_3855* |  | conserved hypothetical protein | 9.54 |
|  | *XC_3856* |  | conserved hypothetical protein | 12.64 |
|  | *XC_3882* |  | conserved hypothetical protein | 8.56 |
|  | *XC_3883* |  | conserved hypothetical protein | 24.34 |
|  | *XC_3961* |  | conserved hypothetical protein | 4.81 |
|  | *XC_3971* |  | conserved hypothetical protein | 9.96 |
|  | *XC_4012* |  | conserved hypothetical protein | 74.96 |
|  | *XC_4013* |  | putative entericidin A | 4.41 |
|  | *XC_4029* |  | conserved hypothetical protein | 2.24 |
|  | *XC_4032* |  | conserved hypothetical protein | 2.09 |
|  | *XC_4033* |  | conserved hypothetical protein | 2.23 |
|  | *XC_4047* |  | conserved hypothetical protein | 2.49 |
|  | *XC_2759* |  | hypothetical protein | 24.42 |
|  | *XC_3968* |  | hypothetical protein | 3.29 |
|  | *XC_3974* |  | hypothetical protein | 10.54 |

Note: False discovery rate (FDR) ≤0.05 and absolute value of log_2_ fold change (log_2_ FC) ≥1 (equivalent to a fold change of 2) were used as the cut off values.“+” represents gene up-regulated in the ∆*vemR* mutant, and “-”represents gene down-regulated.
