# Supplementary material for: Xanthomonas campestris VemR enhances the transcription of the T3SS key regulator HrpX via physical interaction with HrpG
Source: Mol Plant Pathol. 2023 Jan 10;24(3):232–47. doi: 10.1111/mpp.13293 (PMC9923393; doi:10.1111/mpp.13293)
Supplement: Supplementary file 5 — Table S3 Primers used in this work [file MPP-24-232-s002.docx]

**Table S3. Primers used in this study^§^**

| **Primer** | **Nucleotide sequence (5′→3′)** | **The amplified fragment or the utilization** |
| --- | --- | --- |
| L*vemR*-FlagF  L*vemR*-FlagR | CGGGATCCATGAGCAAACTCACCGTGCT  TAGTCGATGTCATGATCTTTATAATCACCGTCATGGTCTTTGTAGTCCTCATTCCTGGCTCCTTCCG | 428-bp DNA fragment containing 381-bp DNA fragmen of *vemR* (*XC_2252*) ORF sequence and 47-bp Flag-coding sequence. Used for constructing *Xcc* strain producing VemR::3×Flag protein. |
| R*vemR*-FlagF  R*vemR*-FlagR | AAAGATCATGACATCGACTACAAGGATGACGATGACAAGTGAGTCCCGCATTCTGTTAA  CCCAAGCTTTTCGGCGTCCAGGCGTTTGA | 415-bp DNA fragment containing 39-bp Flag-coding sequence, 3-bp stop codon and 373-bp downstream of the *vemR* stop codon. Used for constructing *Xcc* strain producing VemR::3×Flag protein. |
| L*xopN*-FlagF  L*xopN*-FlagR | CGGAATTCGTCCGGAACCATGGCTACGG  TAGTCGATGTCATGATCTTTATAATCACCGTCATGGTCTTTGTAGTCTGCCGGTTGCGGCGCCTGAT | 438-bp DNA fragment containing 391-bp DNA fragmen of *xopN* sequence and 47-bp Flag-coding sequence. Used for constructing *Xcc* strain producing *xopN* ::3×Flag protein. |
| R*xopN*-FlagF  R*xopN*-FlagR | AAAGATCATGACATCGACTACAAGGATGACGATGACAAGTGATGCGGCGCCGACCGCGG  CCCAAGCTTCGCGCTACCGCAGCACCTAT | 435-bp DNA fragment containing 39-bp Flag-coding sequence, 3-bp stop codon and 393-bp downstream of the *xopN* stop codon. Used for constructing *Xcc* strain producing *xopN* ::3×Flag protein. |
| L*avrAC*-FlagF  L*avrAC*-FlagR | CGGGATCCGGCATACAAGCTGCCCCTAG  TAGTCGATGTCATGATCTTTATAATCACCGTCATGGTCTTTGTAGTCCTGGTGAACCTGGTTCATAA | 434-bp DNA fragment containing 387-bp DNA fragmen of *avrAC* ORF sequence and 47-bp Flag-coding sequence. Used for constructing *Xcc* strain producing *avrAC* ::3×Flag protein. |
| R*avrAC*-FlagF  R*avrAC*-FlagR | AAAGATCATGACATCGACTACAAGGATGACGATGACAAGTAGACCTACACGCCTCAATG  CCCAAGCTTCTTCCAGGTCGAAGAAGGCC | 456-bp DNA fragment containing 39-bp Flag-coding sequence, 3-bp stop codon and 414-bp downstream of the *avrAC* stop codon. Used for constructing *Xcc* strain producing *avrAC* ::3×Flag protein. |
| L*vemR*-F  L*vemR*-R | GGGGAATTCCGCGACATGCTGCAGGAA   GGGTCTAGAGTTTGCTCATAGTAGTCC | 510-bp DNA sequence upstream of *vemR*, used for constructing the *vemR* deletion mutant. |
| R*vemR*-F  R*vemR*-R | GGGTCTAGAGAATGAGTGAGTCCCGCATTCTG  GGGCTGCAGACCACTTCCTTGCCGGTG | 526-bp DNA sequence downstream of *vemR*, used for constructing the *vemR* deletion mutant. |
| L*hrpG*-F  L*hrpG*-R | GGGGAATTCTCTCACTCTGTCTTGCAAACTGC  GATTCTAGA CATCACTCGCGCGCCCAC | 724-bp DNA sequence upstream of *hrpG*, used for constructing the *hrpG* deletion mutant. |
| R*hrpG*-F  R*hrpG*-R | GAGTCTAGA GCTTGCTGAGCTGCGGCT  GGGCTGCAG ATCATGGTGATCGTGATG | 698-bp DNA sequence downstream of *hrpG*, used for constructing the *hrpG* deletion mutant. |
| *hrpG*-TRGF  *hrpG*-TRGR | CGGGATCCGTGATGGACGCCGCTGCGAAT  CCCTCGAGTCAGCAAGCTGCGGTGCGAT | 789-bp DNA fragment of the *hrpG* (*XC_3077*) ORF sequence. Cloned into target vector pTRG for bacterial two-hybrid assays. |
| *vemR*-BTF  *vemR*-BTR | gggGGATCCAtgagcaaactcaccgtgc  gggctcgagtcactcattcctggctcc | 381-bp DNA fragment of the *vemR* gene coding region. Cloned into the bait vector pBT for bacterial two-hybrid assays. |
| *vemR-*OF  *vemR-*OR | GGGGGATCC ATGAGCAAACTCACCGTG  GGGAAGCTT TCACTCATTCCTGGCTCC | 381-bp DNA fragment of the *vemR* gene coding sequence. Used for overexpression and pull-down assays. |
| *hrpG-*OF  *hrpG*-OR | GGGGAATTCGTGATGGACGCCGCTGCG  GGGCTCGAG TCAGCAAGCTGCGGTGCG | 789-bp DNA fragment of the *hrpG* (*XC_3077*) ORF sequence. Used for overexpression and pull-down assays. |
| *mcvR-*OF  *mcvR*-OR | GGGGGATCCTTGCGTGGAGTTCGGGTA  GGGAAGCTTTCAGCTGCTGGCACCGCT | 369-bp DNA fragment of the *mcvR* (*XC_1966*) coding sequence.Used for overproduction and pull-down assays. |
| *hupB*-OF  *hupB-*OR | GGGGATCCATGAATAAAACCGAATTG  GCGGAAGCTTTTAGTTTACTGCATCCTT | 270-bp DNA fragment of the *hupB* (*XC_3262*) ORF sequence. Used for overexpression and pull-down assays. |
| 16SF  16SR | GCCTAACACATGCAAGTCGAACGGC  AATATTCCCCACTGCTGCCTCCCG | 325-bp DNA fragment of the 16S rDNA sequence, used for RT-PCR and qRT-PCR. |
| *avrAC*-F  *avrAC*-R | CGGCAGATCATCCAACGCAC  CCGCGTTTCCTGAAGGCTTC | 116-bp DNA fragment spans nucleotides 62 to 177 bp of the *avrAC*, used for qRT-PCR. |
| *xopN*-F  *xopN*-R | AATCCGCCCAGTCGCACTGT  TTGTTCGGGCGGCTGGATAG | 140-bp DNA fragment spans nucleotides 64 to 203 bp of the *xopN*, used for qRT-PCR. |
| *hrpG*-F  *hrpG*-R | AAGAAACTGCGGCTGTGC  GGTGCGATTGACCGTATTG | 144-bp DNA fragment spans nucleotides 634 to 777 bp of the *hrpG*, used for qRT-PCR. |
| *hrpX*-F  *hrpX*-R | GAGACATCTTCGGCTTCG  GCCTGGCAATACTCGAAC | 205-bp DNA fragment spans nucleotides 746 to 950 bp of the *hrpX*, used for qRT-PCR. |
| *hrcN*-F  *hrcN*-R | TTGCAGGCGACGATTGGGTG  TTGACATCGCACTGCGTGCC | 211-bp DNA fragment spans nucleotides 365 to 575 bp of the *hrcN*, used for qRT-PCR. |
| *hrcU*-F  *hrcU*-R | TGGTGATGAAGCTGGCAGGC  AGGTGCCGATCAATCCTGCG | 192-bp DNA fragment spans nucleotides 119 to 310 bp of the *hrcU*, used for qRT-PCR. |
| *hrpF*-F  *hrpF*-R | CAATCAGCACAAAGACAAGACGC  AAATCACCCGCCTTGATCTTG | 183-bp DNA fragment spans nucleotides 414 to 596 bp of the *hrpF*, used for qRT-PCR. |
| *hrcQ*-F  *hrcQ*-R | CCTCGATATCGGCATCACGC  GAGCGTGCAACAGGACATCG | 227-bp DNA fragment spans nucleotides 288 to 512 bp of the *hrcQ*, used for qRT-PCR. |
| *hrcC*-F  *hrcC*-R | TCAGAATCTGGGGCGCGAAC  TATCCACATAACCCGGCGGG | 177-bp DNA fragment spans nucleotides 323 to 499 bp of the *hrcC*, used for qRT-PCR. |
| *hrpB2*-F  *hrpB2*-R | TGTACCGATCACTGCAAGTACCG  TCGTTCTGCATGTCCACCATG | 180-bp DNA fragment spans nucleotides 21 to 200 bp of the *hrpB2*, used for qRT-PCR. |
| *hrpX*ivt-F  *hrpX*ivt-R | CGCATCGTCTGGCCTTCGTC  TTGCTGGAGGTGCTGCAGACCCTGT | 311-bp DNA fragment of *hrpX* spanning nucleotides from -131 to +179 relative to TIS, used for in vitro transcription assay. |
| 2239-F  2239-R | CTGTATCAACAGGTGCGCGC  CGTTGACCATCACGTCGAGC | 163-bp DNA fragment spans nucleotides 133 to 295 bp of the *flgG*, used for qRT-PCR. |
| 2265-F  2265-R | ATGCTCAACCCGGCCAACAC  TCCTTGGTGGACTGCTTGGC | 209-bp DNA fragment spans nucleotides 118 to 326 bp of the *fliK*, used for qRT-PCR. 3.03 |
| 2267-F  2267-R | ACCCTGGAGATGGTCAACGAG  GGATCAGGTTGAGGTTGGTGG | 160-bp DNA fragment spans nucleotides157 to 316 bp of the *fliM* used for qRT-PCR. |
| 0940-F  0940-R | GATCTGGTTCTACTACGACGGGC  GCTCCTCGATGACCTTCTTGC | 158-bp DNA fragment spans nucleotides 114 to 271 bp of the *pilN*, used for qRT-PCR. |
| 1183-F  1183-R | ATGACTGAAAACATGGCTGCG  AAATGATCTGAGGTTGCTGGTCC | 184-bp DNA fragment spans nucleotides 1 to 184 bp of the *pilG*, used for qRT-PCR. |
| 1358-F  1358-R | CGCCGATCTTTCGCCAGTTG  TTCGCGCTGGTTGATCAGGC | 197-bp DNA fragment spans nucleotides 335 to 531 bp of the *pilT*, used for qRT-PCR. |
| 1409-F  1409-R | GGACGTGATCGTGCTGGATG  AACTGCTTGAGTCCCAGCCG | 195-bp DNA fragment spans nucleotides 132 to 326 bp of the *cheB*, used for qRT-PCR. |
| 1409-F  1409-R | GGACGTGATCGTGCTGGATG  AACTGCTTGAGTCCCAGCCG | 195-bp DNA fragment spans nucleotides 132 to 326 bp of the *cheB*, used for qRT-PCR. |
| 1222-F  1222-R | GAAGGCGAAAAGGTCAGCATC  CTCCATCAGGCCGATGATTTC | 165-bp DNA fragment spans nucleotides 316 to 480 bp of the *iroN*, used for qRT-PCR. |
| 2485-F  2485-R | TGGACCTGGACACAGTGGAAGTG  CATGAAGCGGTCGATGGTCAC | 182-bp DNA fragment spans nucleotides 161 to 342 bp of the *fhuA*, used for qRT-PCR. |
| 0741-F  0741-R | CGGCAAGGACCTGGTGTTGTTC  ACCAATGCATGCGTCTGGCC | 141-bp DNA fragment spans nucleotides 171 to 311 bp of the *xcsF*, used for qRT-PCR. |
| 0783-F  0783-R | CCGGGCCGTACAAGATCTTC  GGGGTTCCAGTCGTAATGCC | 188-bp DNA fragment spans nucleotides 131 to 318 bp of the *celS*, used for qRT-PCR. |
| 1638-F  1638-R | AAAGATGTGCTTTTCCGTGGGTG  GCCACTTGCTGCATGACGAAG | 164-bp DNA fragment spans nucleotides 7 to 170 bp of the *virB3*, used for qRT-PCR. |
| 2016-F  2016-R | CATGGAGTTGATGAGCCGCG  CGACCTTGCCGGCTTTGATC | 149-bp DNA fragment spans nucleotides 123 to 271 bp of the *virB6*, used for qRT-PCR. |

^§^The underlined sequences indicate the restriction sites for *Bam*HI, *Eco*RI, *Hin*dIII, *Kpn*I, *Pst*I, *Sac*I, *Xba*I and *Xho*I, respectively. The long square boxes indicate the Flag-coding sequences, and highlights in gray show the complementary sequences.
