# Supplementary material for: Xanthomonas campestris VemR enhances the transcription of the T3SS key regulator HrpX via physical interaction with HrpG
Source: Mol Plant Pathol. 2023 Jan 10;24(3):232–47. doi: 10.1111/mpp.13293 (PMC9923393; doi:10.1111/mpp.13293)
Supplement: Supplementary file 6 — Table S4 The potential VemR‐interacting proteins identified by co‐immunoprecipitation coupled with LC–MS/MS assays [file MPP-24-232-s001.docx]

**Table S4** The potential VemR-interacting proteins identified by Co-IP coupled with LC-MS/MS assays

| **Gene ID** | **Name** | **Annotation** | **FDA (Exp. q-value)** |
| --- | --- | --- | --- |
| *XC_0008* |  | TonB protein | 0 |
| *XC_0063* |  | Regulatory protein cII | 0 |
| *XC_0438* |  | ATP-dependent RNA helicase | 0.003012048 |
| *XC_0638* |  | Chemotaxis protein | 0 |
| *XC_0718* |  | NMN adenylyltransferase | 0 |
| *XC_0729* |  | Two-component system regulatory protein | 0 |
| *XC_0984* |  | Transcriptional regulator lysR family | 0 |
| *XC_0994* |  | ATP sulfurylase/adenylylsulfate kinase | 0 |
| *XC_1060* |  | Pilus biogenesis protein | 0 |
| *XC_1061* | *pilR* | Two-component system regulatory protein PilR | 0 |
| *XC_1064* |  | Succinyl-CoA ligase [ADP-forming] subunit alpha | 0 |
| *XC_1139* | *tolR* | biopolymer transport protein TolR | 0 |
| *XC_1186* | *pilJ* | Pilus biogenesis protein | 0.006116208 |
| *XC_1187* | *pilL* | PilL protein | 0 |
| *XC_1261* |  | Histidine kinase/response regulator hybrid protein | 0.019417476 |
| *XC_1307* | *hpaP* | Nucleotide-binding protein | 0 |
| *XC_1308* | *hprK* | HPr kinase/phosphorylase | 0 |
| *XC_1346* |  | ABC transporter ATP-binding protein | 0.00511509 |
| *XC_1358* |  | Twitching motility protein | 0.029325513 |
| *XC_1359* |  | Twitching motility protein | 0 |
| *XC_1372* |  | Diaminopimelate decarboxylase | 0 |
| *XC_1475* |  | ATP-dependent RNA helicase DeaD | 0 |
| *XC_1591* | *nuoC* | NADH-quinone oxidoreductase subunit C | 0.00511509 |
| *XC_1594* |  | NADH-ubiquinone oxidoreductase NQO1 subunit | 0 |
| *XC_1595* |  | NADH-ubiquinone oxidoreductase NQO3 subunit | 0 |
| *XC_1616* |  | Glutaredoxin Hypothetical protein | 0.00511509 |
| *XC_1625* | *pilY1* | PilY1 protein | 0 |
| *XC_1678* | *dxs* | 1-deoxy-D-xylulose-5-phosphate synthase | 0 |
| *XC_1824* |  | Uncharacterized protein | 0 |
| *XC_1933* | *folD* | ethylenetetrahydrofolate dehydrogenase (NADP+) | 0 |
| *XC_2147* |  | Hydroxylase | 0 |
| *XC_2160* |  | YapH protein | 0.00511509 |
| *XC_2229* | *ravA* | two-component system sensor histidine kinase RavA | 0 |
| *XC_2243* | *FlgK* | Flagellar protein | 0.020958084 |
| *XC_2245* |  | Flagellin | 0 |
| *XC_2266* | *fliL* | Flagellar biosynthesis protein | 0.00511509 |
| *XC_2267* | *fliM* | Flagellar motor switch protein FliM | 0 |
| *XC_2268* | *fliN* | Flagellar motor switch protein FliN | 0 |
| *XC_2282* | *cheY* | Chemotaxis protein CheY | 0.003012048 |
| *XC_2284* | *cheA* | Chemotaxis related protein CheA | 0.006116208 |
| *XC_2304* |  | Chemotaxis protein | 0.031518625 |
| *XC_2311* |  | Chemotaxis protein | 0 |
| *XC_2346* |  | Carbamoyl-phosphate synthase (glutamine-hydrolyzing) | 0 |
| *XC_2470* |  | Polynucleotide adenyltransferase | 0 |
| *XC_2718* |  | Ribonuclease R | 0 |
| *XC_2723* |  | Transcriptional regulator | 0.028089888 |
| *XC_2737* |  | ABC transporter ATP-binding protein | 0 |
| *XC_2740* |  | Uncharacterized protein | 0 |
| *XC_2762* | *dnaJ* | Chaperone protein DnaJ | 0 |
| *XC_2767* |  | Ferric uptake regulator Fur | 0 |
| *XC_2801* |  | Transcriptional regulator | 0.006116208 |
| *XC_2824* |  | Ferredoxin-NADP reductase | 0.007389163 |
| *XC_2842* |  | DNA topoisomerase 4 subunit A | 0 |
| *XC_2864* | *tsf* | Elongation factor Ts | 0.003012048 |
| *XC_2971* | *lepA* | Elongation factor 4 | 0 |
| *XC_3067* |  | Histidine kinase/response regulator hybrid protein | 0 |
| *XC_3072* | *rsbR* | Positive regulator of sigma-B | 0.003968254 |
| *XC_3077* | *hrpG* | Transcriptional regulator HrpG | 0 |
| *XC_3083* |  | Cytochrome O ubiquinol oxidase subunit I | 0 |
| *XC_3227* |  | 3-oxoacyl-[ACP] reductase | 0 |
| *XC_3261* |  | Peptidyl-prolyl cis-trans isomerase | 0 |
| *XC_3313* |  | Family II 2-keto-3-deoxy-D-arabino-heptulosonate 7-phosphate synthase | 0 |
| *XC_3343* | *fusA* | Elongation factor G | 0 |
| *XC_3347* | *rpoB* | DNA-directed RNA polymerase subunit beta | 0 |
| *XC_3505* | *ftsZ* | Cell division protein FtsZ | 0 |
| *XC_3523* |  | Uncharacterized protein | 0 |
| *XC_3597* | *hns* | DNA-binding protein |  |
| *XC_3612* |  | Glucose-1-phosphate thymidylyltransferase | 0 |
| *XC_3618* |  | Glycosyltransferase | 0 |
| *XC_3621* |  | Uncharacterized protein | 0 |
| *XC_3629* | *wxcD* | Membrane protein WxcD | 0 |
| *XC_3640* | *pilU* | Type II secretion system protein-like protein | 0 |
| *XC_3652* |  | Beta-ketoacyl-[ACP] synthase I | 0 |
| *XC_3670* | *hpaS* | Two-component system histidine kinase HpaS | 0 |
| *XC_3674* | *glmU* | UDP-N-acetylglucosamine pyrophosphorylase GlmU | 0 |
| *XC_3681* | *atpH* | ATP synthase subunit delta | 0 |
| *XC_4031* |  | Two-component system regulatory protein | 0 |
| *XC_4076* |  | Ribonucleoside-diphosphate reductase | 0 |
| *XC_4130* | *rsmG* | Ribosomal RNA small subunit methyltransferase G | 0 |
